# Further Biochemical Profiling of *Hypholoma fasciculare* Metabolome Reveals Its Chemogenetic Diversity

**DOI:** 10.3389/fbioe.2021.567384

**Published:** 2021-05-24

**Authors:** Suhad A. A. Al-Salihi, Ian D. Bull, Raghad Al-Salhi, Paul J. Gates, Kifah S. M. Salih, Andy M. Bailey, Gary D. Foster

**Affiliations:** ^1^School of Biological Sciences, University of Bristol, Bristol, United Kingdom; ^2^School of Chemistry, University of Bristol, Bristol, United Kingdom; ^3^Chemistry Department, University of Mustansiriyah, Baghdad, Iraq; ^4^College of Arts and Sciences, Qatar University, Doha, Qatar

**Keywords:** *Hypholoma fasciculare*, genome, metabolome, terpene, biosynthetic gene cluster

## Abstract

Natural products with novel chemistry are urgently needed to battle the continued increase in microbial drug resistance. Mushroom-forming fungi are underutilized as a source of novel antibiotics in the literature due to their challenging culture preparation and genetic intractability. However, modern fungal molecular and synthetic biology tools have renewed interest in exploring mushroom fungi for novel therapeutic agents. The aims of this study were to investigate the secondary metabolites of nine basidiomycetes, screen their biological and chemical properties, and then investigate the genetic pathways associated with their production. Of the nine fungi selected, *Hypholoma fasciculare* was revealed to be a highly active antagonistic species, with antimicrobial activity against three different microorganisms: *Bacillus subtilis*, *Escherichia coli*, and *Saccharomyces cerevisiae*. Genomic comparisons and chromatographic studies were employed to characterize more than 15 biosynthetic gene clusters and resulted in the identification of 3,5-dichloromethoxy benzoic acid as a potential antibacterial compound. The biosynthetic gene cluster for this product is also predicted. This study reinforces the potential of mushroom-forming fungi as an underexplored reservoir of bioactive natural products. Access to genomic data, and chemical-based frameworks, will assist the development and application of novel molecules with applications in both the pharmaceutical and agrochemical industries.

## Introduction

Mushroom-forming fungi are recognized for producing a plethora of chemical compounds to help defeat competitive organisms that coexist in their ecosystem (Schmidt-Dannert, 2016). These are specialized secondary metabolites (SMs) or natural products, which are known for their wide-ranging useful biological activities including antimicrobial, antitumor, and insecticide properties (Wilkins and Harris, 1944; Wilkins, 1946; de Mattos-Shipley et al., 2016). Over the recent decades, antibiotic drug discovery has mostly focussed on bacteria and ascomycetes fungi, overlooking the potential of basidiomycetes (Robbins et al., 1947). Reports in the literature state that only 20% of the current antibiotics are of fungal origin (Newman and Cragg, 2016). This is most likely because fungi primarily produce their SMs to outcompete their competitors in their environment and that many of these triggers or cues are not reproducible under laboratory conditions (Jakubczyk and Dussart, 2020). Compared to other groups of fungi, mushroom-forming basidiomycetes grow at a slow rate and reproduce using dikaryotic cell types, making genetic investigations more complex and time-consuming. Therefore, it was predicted that these basidiomycetes may produce exploitable novel compounds (Rahi and Malik, 2016). Although basidiomycetes are known to produce mycotoxins with significant biological activities with both medicinal and agricultural applications, for example psilocybin from *Psilocybe* spp., strobilurin from *Strobilurus tenacellus*, and pleuromutilin from *Clitopilus passeckerianus* (Bailey et al., 2016; Nofiani et al., 2018; Fricke et al., 2019), the main groups of natural products that have been isolated from basidiomycetes fungi are typically halogenated compounds and terpenoids. These include tetrachlorinated phenols, illudanes, sterpurenes, and illudalanes, all of which are largely produced exclusively by fungi, with one exception being illudalanes, which are also produced by some plants (De Jong and Field, 1997; Quin et al., 2014).

Given the number of unstudied Agaricales species, there is a high probability of finding SMs with useful biological activities, such as antibiotics, within this group of organisms. *H. fasciculare* (*Naematoloma fasciculare*) is an inedible mushroom-forming basidiomycete found on decaying wood. It is commonly named “sulfur tuft” due to its growth pattern (tight clusters or tufts of mushrooms) and the bright sulfur yellow color of its cap. Several field studies have shown the capability of this fungus to control the colonization of other wood decay organisms (De Jong et al., 1994). *H. fasciculare* has previously been reported in the literature as a rich source of terpenoid and organohalogen natural products including fascicularones and anisaldehyde metabolites; however, the pharmaceutical properties and the biological synthesis of these SMs remain unstudied (Supplementary Figure 1 lists all chemicals characterized from *H. fasciculare* from 1967 to 2019). To gain a greater understanding of the biogenetics and biochemistry of the *H. fasciculare* metabolome, a series of genetic manipulations, bioactivity assays, and chemical analyses of crude and pure extracts of *H. fasciculare* were performed to detect any potential antimicrobial activities and to further investigate the metabolic potential of this fungus. The overall aim was to attempt to bridge the gap between natural bioactive molecules and combating antibacterial resistance.

## Materials and Methods

### *H. fasciculare* Genome Mining

Our previous genomic investigation of *Hypholoma* suggested that only terpenoid compounds were produced, with a range of cyclization patterns (Al-Salihi et al., 2019). However, a subsequent in-depth BLAST search of functionally characterized core enzymes selected from different fungi resulted in the identification of additional biosynthetic gene clusters (BGCs) in both *Hypholoma* species (see Supplementary Tables 1, 2). The introns and exons of selected scaffolds were predicted using a combination of Softberry and Local BLAST searches, allowing the subsequent functional analysis of the predicted biosynthetic gene clusters. Annotation of the predicted open reading frames of each of the submitted contigs was then carried out using Artemis. Protein BLAST search on NCBI was undertaken for each gene for possible function prediction. At least 10 genes either side of the predicted SM core enzymes were annotated. The *Hypholoma* BGCs were then manually curated by BLAST searches against the *Hypholoma sublateritium* genome on JGI.

### Antisense Plasmid Construction and *Agrobacterium* Transformation

#### Construction of Antisense Vector Targeting Argininosuccinate Synthetase Gene

Argininosuccinate synthetase is an essential protein for fungal growth, and successful silencing would present as a starved growth pattern, allowing a quick and simple assessment of any potential silencing. The published sequences of *H. sublateritium* argininosuccinate synthetase were BLAST searched against the *H. fasciculare* genome. A gene with 93% identity was identified in *H. fasciculare* contig 63. The argininosuccinate antisense plasmid consisted of a pCAMBIA0380YA backbone, 500 bp of the *H. fasciculare* argininosuccinate gene, which was inserted (in the antisense orientation) between the *H. sublateritium gpd* promoter and TrpC terminator, and the hygromycin cassette (*hph* gene under the *Agaricus bisporus gpd*II promoter and CaMV35S terminator). The verified argininosuccinate synthetase-silencing construct was moved into the *Agrobacterium tumefaciens* strain LBA4404 and used to transform *H. fasciculare* (Supplementary Table 3 lists the primers used for the construction and confirmation of the antisense plasmids).

#### Silencing of *H. fasciculare* Terpene Synthases

During the *in silico* analysis, it appeared that terpene synthases are the most common *H. fasciculare* SM gene clusters. RNA interference (RNAi)-mediated gene silencing of the core terpene synthases was performed in an attempt to link each predicted terpene synthase gene to at least one of the previously reported natural molecules from *H. fasciculare*. Genes were selected according to the predicted enzymatic carbon cyclization pattern, including five representatives predicted to exhibit 1,11 carbon cyclization (HfasTerp-255, HfasTerp-94A, HfasTerp-94B, and HfasTerp-105). The remaining genes were as follows: HfasTerp-147 for 1,10 3RNNP, HfasTerp-804 for 1,6 3R/S-NPP, and HfasTerp-342 and HfasTerp-179 for 1,10, E,E,farnesyl diphosphate (E,E-FPP). The atypical HfasTerp-85b was also included in this investigation.

Prior to antisense plasmid construction, reverse transcription PCR (RT-PCR) was deployed for the genes selected to confirm their predicted splicing patterns. The introns of all nine selected terpene synthases plus two housekeeping genes (*gpd* and β-*tubulin*) were predicted using a combination of SoftBerry and Artemis. RNA was extracted from growing mycelial cultures, in which their antimicrobial activity had already been confirmed. Complementary DNAs (cDNAs) were then synthesized with Oligo(dT)18 primers, and amplification of 150- to 250-bp fragments spanning at least one intron was carried out for each gene. These cDNA-derived segments of terpene synthase genes were cloned into silencing vectors as described above.

#### Chemical Profiling of *H. fasciculare* Silenced Lines

Mycelial plugs of the silenced transformants were individually inoculated into 100 ml of MEB (15 g/L malt extract broth) in a 250-ml flask and incubated at 25°C and 200 rpm for 21 days. The previously described ethyl acetate metabolite extraction protocol was used (Bailey et al., 2016). The chemical compositions of the wild type and the silenced lines (20 μl, final concentration of 5 mg/ml) of each crude extract were then compared by high-performance liquid chromatography (HPLC) as described in (Al-Salihi et al., 2019).

#### Expression of Selected Terpene Synthase Enzymes in *Aspergillus oryzae*

To avoid the potential issue associated with intron miss-splicing, full-length cDNA templates for the selected genes (HfasTerp-94A, HfasTerp94B, HfasTerp179, and HfasTerp344) were synthesized by RT-PCR. The cDNA versions of the sesquiterpene synthases (Cop-1, Cop-2, Cop-3, Cop-4, Omph-6, and Omph-7) were kindly provided by Schmidt’s group (Agger et al., 2009; Wawrzyn et al., 2012). Expression vectors were generated by yeast-based recombination as described in Al-Salihi et al. (2019). *A. oryzae* transformants were generated for the 10 selected enzymes and chemically analyzed using the protocol described in Al-Salihi et al. (2019).

## Results

### Bioassay

**FIGURE 1:**
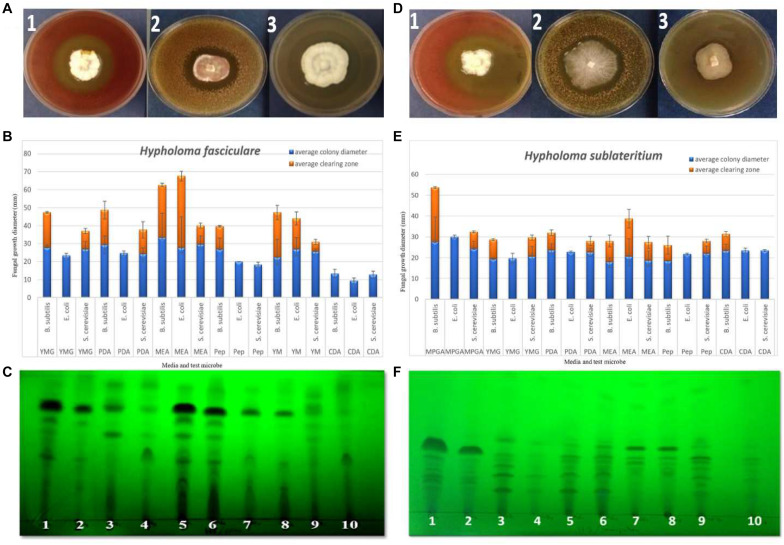
**(A,D)** Examples of the zone inhibition plates of *Hypholoma fasciculare* and *Hypholoma sublateritium* showing the clearing zone around the fungal colony, indicating the antimicrobial activity of these fungi against *Bacillus subtilis (1), Saccharomyces cerevisiae (2), and Escherichia coli (3)*, respectively. **(B)** Zone inhibition assay to evaluate the antimicrobial activity of *H. fasciculare* growing on different media against *B. subtilis*, *E. coli*, and *S. cerevisiae*. *Error bars* indicate the standard deviations of three technical replicate measurements for both fungal colony diameter (column in *blue*) and inhibition zone diameter (column in *red*). **(E)** Zone inhibition assay of *H. sublateritium* growing on different media against *B. subtilis*, *E. coli*, and *S. cerevisiae*. *Error bars* indicate the standard deviations of three technical replicate measurements. **(C,F)** Thin-layer chromatography (TLC) plates developed in a polar (*H. fasciculare*) and a semi-polar (*H. sublateritium*) system and visualized under UV 320 nm showing the separated compounds of *H. fasciculare*. Twenty-five microliters of 10 mg/ml crude extract of *H. fasciculare* from five different media was spotted. *1* = CSO supernatant extract; *2* = CSO mycelial extract; *3* = PDB supernatant extract; *4* = PDB mycelial extract; *5* = YMG supernatant extract; *6* = YMG mycelial extract; *7* = CGC supernatant extract; *8* = CGC mycelial extract; *9* = MEB supernatant extract; *10* = MEB mycelial extract. *YMG*, yeast extract malt; *PDB*, potato dextrose broth; *PDA*, potato dextrose agar; *MEA*, malt extract agar; *Pep*, peptone agar; *YM*, yeast malt extract; *CDA*, Czapek Dox agar; *MPGA*, malt peptone glucose agar.

We assayed nine basidiomycetes to determine their ability to produce bioactive SMs on a range of solid media (see Supplementary Material for details of the method), from which the two Strophariaceae species (*H. fasciculare* and *H. sublateritium*) displayed noticeable antimicrobial activity against the three challenged microbes (see Figure 1). In contrast, *Paxillus involutus* showed no activity against any of the microbes tested. Variable inhibition zones were produced by the remaining basidiomycetes (see Supplementary Figures 2–7 for media, test microbes, and clearing zones diameter description).

### Bioautography

Due to the broad similarity between the chemical profiles of the crude extracts of both the mycelia and supernatant of the five culture media tested for both *Hypholoma* strains (data not shown), it was decided to use homogenized cultures for the extraction of the metabolites. The chemical extracts were then separately spotted on the thin-layer chromatography (TLC) plates and developed in different solvent systems—non-polar, polar, and semi-polar—which were then tested against *Bacillus subtilis* (see Supplementary Figures 9–12 for TLC plate examples).

The antibacterial activity of the crude extracts separated using a polar system showed one large zone of inhibition (sizes ranged from 20 to 26 mm), and the highest antibacterial activity (26 mm) was observed for the crude extracts of yeast extract malt (YMG) among the other extracts of *H. fasciculare* and in potato dextrose broth (PDB, 25 mm) crude extract of *H. sublateritium*. Supplementary Figures 13–15 show some examples of the bioautography plates of *H. fasciculare.*

### Chemical Profiling and Structure Elucidation of 3,5-Dichloro-4-Methoxybenzoic Acid

Analysis of the bioautography assays suggested similar chemical profiles for both *Hypholoma* species. As a result, the metabolites *H. fasciculare* species were purified. The variations in the retention factors (RFs) of the observed active compounds in the bioautography plates suggested that they were different metabolites. CSO-1A, YMG, CGC, and MEB were then selected for purification. Initial HPLC analysis of the crude extracts was performed to select the most potent samples for metabolite purification. This led to the detection of five main products: A, B, C, D, and E (Supplementary Figure 16). Subsequent isolation experiments were attempted to purify the five major peaks.

**FIGURE 2:**
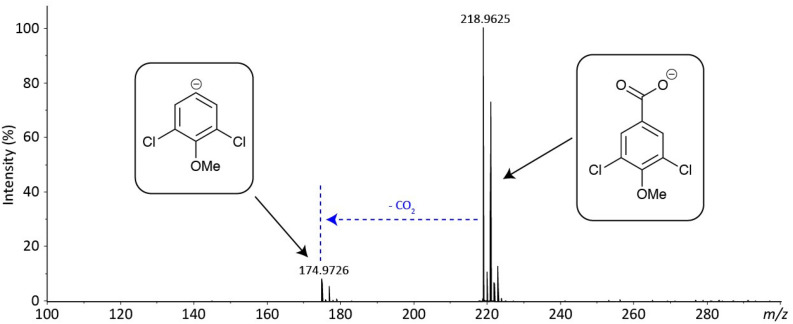
Accurate-mass negative ion electrospray ionization (ESI) mass spectrum of fraction C—putative 3,5-dichloro-4-methoxy benzoic acid. The spectrum shows the [M-H]^–^ at *m*/*z* 218.9625 (218.9621 for C_8_H_5_O_3_Cl_2_^–^) and fragmentation resulting from decarboxylation at *m*/*z* 174.9726 (174.9723 for C_7_H_5_OCl_2_^–^).

Despite attempting different fractionation programs, only two fractions (A and D) could be purified from the crude extracts. A comparison was then carried out between the mass spectra of the purified metabolites (A and D) and the compounds previously reported from the *Hypholoma* genus, leading to the characterization of metabolite A as fascicularone G (a known sesquiterpene from *Hypholoma*). However, for fraction D, no match could be found with any of the previously characterized metabolites from *Hypholoma*. We therefore subjected fraction D to high-resolution mass spectrometry (HRMS) to confirm its exact mass and predict its molecular formula (see Figure 2). This analysis indicated an aromatic molecule of chemical formula C_8_H_6_O_3_Cl_2_ to better than 2 ppm mass accuracy. This is putatively assigned as 3,5-dichloro-4-methoxybenzoic acid (3,5-D). The isotope distribution of the [M-H]^–^ ion also matches the predicted distribution. The only fragment ion in the spectrum is due to decarboxylation (loss of CO_2_), which is typical of an aromatic carboxylic acid in negative ion mode, which provides further evidence for the assignment of metabolite D.

The structure of compound D was further investigated using ^1^H and ^13^C NMR, where the presence of a symmetrical benzoic acid molecule, two chlorines, and one methoxy group was confirmed. The correlations between the aromatic protons at 7.9 and 3.8 ppm and the carbon signals were also investigated by heteronuclear multiple bond correlation (HMBC), and the structure of 3,5-dichloro-4-methoxybenzoic acid was confirmed (see Supplementary Figures 17–21 for the NMR characterization).

### Antimicrobial Activity of 3,5-D

The antibacterial activity of 3,5-D was assessed by disk diffusion assay using gradient concentrations of 100, 300, and 500 μg. Disks containing methanol and kanamycin were used as negative and positive control, respectively. As shown in Table 1, the antibacterial activity of 3,5-D increased with an increase of its concentration against *Staphylococcus aureus* and *B. subtilis*.

**TABLE 1:** Antimicrobial activity of 3,5-dichloro-4-methoxybenzoic acid (3,5-D) tested against *Bacillus subtilis* and *Staphylococcus aureus.*

**Microorganism**	**Clearing zone (mm) disk diameter: 5 mm Average clearing zone (mm) ± SD of 3 technical replicates (disc diameter 5mm)**				
	**100 μg/disk kanamycin (positive control)**	**100 μl of 98% methanol (negative control)**	**100 μg/disk of 3,5-D**	**300 μg/disk of 3,5-D**	**500 μg/disk of 3,5-D**
*B. subtilis*	12 ± 0	–	12 ± 0.7	16 ± 0	20 ± 0.5
S. aureus^*a*^	11	–	10	14	20

**^*a*^The clearing zone results represent one replicate.**

### Genomic Investigation

Analysis of the *H. fasciculare* genome using FungiSmash identified 15 pairs of orthologous BGCs for *H. fasciculare* and *H. sublateritium*, as outlined below (detailed description of all predicted BGCs can be found in Supplementary Figures 22–30).

**FIGURE 3:**
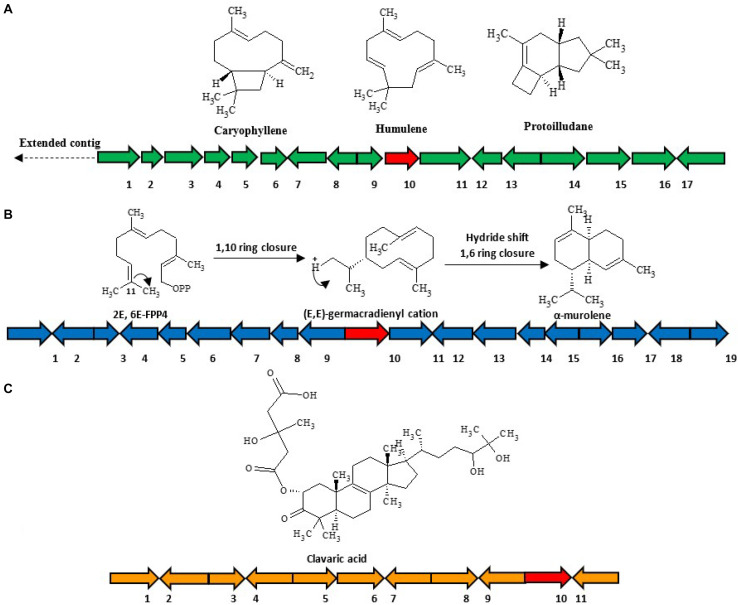
Selected sesquiterpene biosynthetic gene clusters of 1,11 E,E-FPP carbon cyclization clade identified in the *Hypholoma fasciculare* genome and their likely initial products. **(A)** HfasTerp-94a and HfasTerp-94b biosynthetic gene clusters and examples of likely products—caryophyllene, humulene, and protoilludane—illustrating the 1,11 carbon cyclization pattern predicted for this synthase. *1* = Serine/threonine kinase; *2* = Zinc carboxypeptidase; *3* = Hypothetical protein; *4* = Short-chain dehydrogenase 1; *5* = Short-chain dehydrogenase 2; *6* = Short-chain dehydrogenase 3; *7* = Tyrosinase; *8* = F-box domain; *9* = Anchor signaling protein; *10* = Terpene synthase B; *11* = Splicing co-activator; *12* = F-box domain; *13* = Glucose transporter; *14* = Peptide transporter; *15* = Terpene synthase A; *16* = Aromatic ring hydroxylase; *17* = Glycoside hydrolase. **(B)** HfasTerp-179 biosynthetic gene cluster (BGC) and examples of likely products arising from the predicted 1,10 carbon cyclization for this synthase. *1* = Alpha/beta-hydrolases; *2* = Aspartyl protease; *3* = E3 ubiquitin–protein ligase; *4* = NAD-dependent histone deacetylase; *5* = Hypothetical protein; *6* = Transcription factor; *7* = GMC oxidoreductase; *8* = Hypothetical protein; *9* = Cytochrome P450; *10* = Terpene synthase; *11* = GMC oxidoreductase; *12* = Fructose 2,6-bisphosphatase; *13* = Kinase like protein; *14* = Hypothetical protein; *15* = DNA repair endonuclease; *16* = Telomerase transcriptase; *17* = Monooxygenase FAD; *18* = Glycoside hydrolase; *19* = Alpha ketogluturate. **(C)** HfasTerp-804 BGC predicted to be responsible for the production of the antitumor clavaric acid or similar derivatives. *1* = Hypothetical protein; *2* = Flavin-containing amine oxidase; *3* = Pyruvate decarboxylase; *4* = Cytoplasmic protein; *5* = Phosphomannomutase; *6* = Delta DNA polymerase; *7* = Putative transcription factor; *8* = 60S ribosomal protein translation; *9* = Actin-related protein; *10* = Oxidosqualene clavarinone cyclase; *11* = Nuclear condensin complex.

**FIGURE 4:**
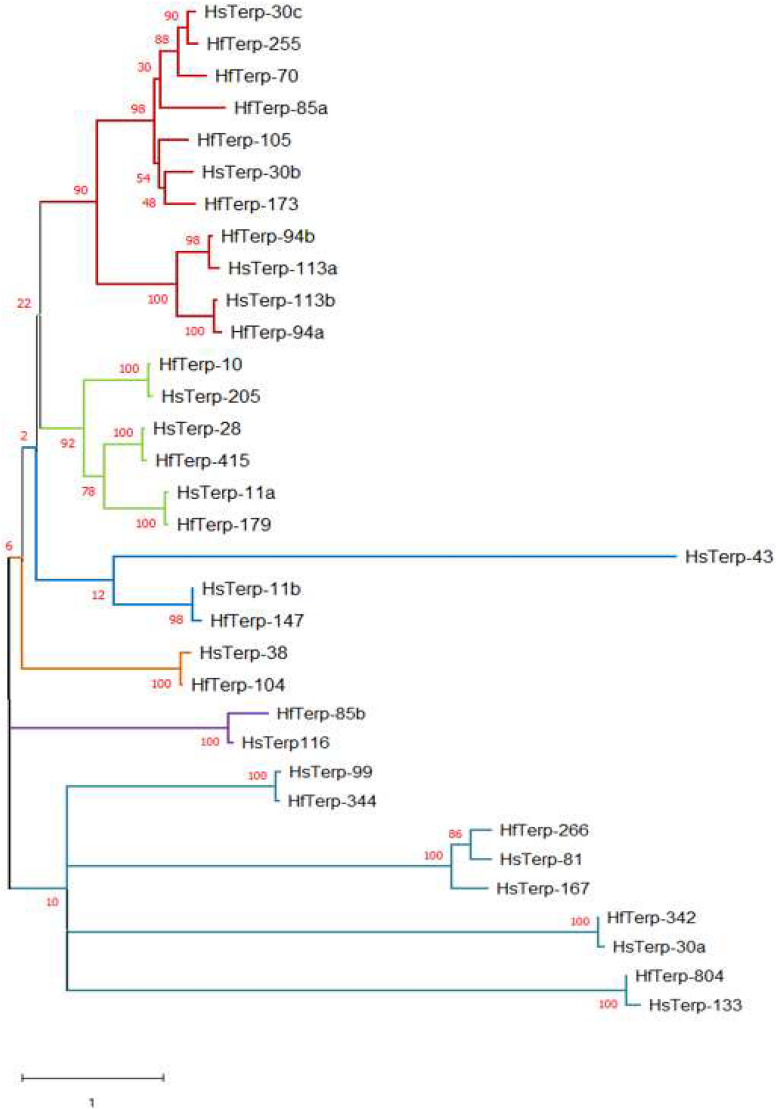
Maximum likelihood tree of putative terpene synthases from *Hypholoma fasciculare* and *Hypholoma sublateritium*. Contigs or scaffold numbers are shown *adjacent to species abbreviations*. The evolutionary history was inferred by using the maximum Likelihood method and the Whelan and Goldman model conducted in MEGA X. The percentages of trees in which the associated taxa clustered together are shown *next to the branches*.

**FIGURE 5:**
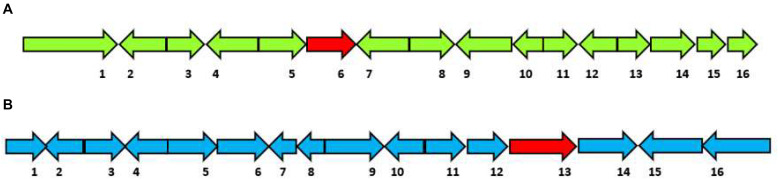
Schematic illustration of the organization of the putative biosynthetic gene clusters for both HfasTerp-147 and HfasTerp-104. **(A)** The extended HfasTerp-147 biosynthetic gene cluster. *1* = Complex I intermediate-associated protein; *2* = Protein binding; *3* = F-box; *4* = Histone methyltransferase; *5* = RNA polymerase; *6* = Terpene synthase; *7* = Cystathionine beta-lyase; *8* = Pyruvate carboxyltransferase; *9* = Dolichol kinase; *10* = Zinc finger; *11* = Splicing factor; *12* = Proliferation-associated protein; *13* = Chaperone regulator; *14* = Enoyl-CoA hydratase; *15* = Aspartic peptidase; *16* = Aspartic peptidase. **(B)** Organization of the putative biosynthetic gene cluster likely responsible in producing 3,5-dichloro-4-methoxybenzoic acid (3,5-D) in both *Hypholoma fasciculare* and the homologous cluster of *Hypholoma sublateritium*. *1* = Glycoside; *2* = Glutathione S-transferase; *3* = Anchor protein; *4* = Oxoprolinase; *5* = Monocarboxylate; *6* = Glycoside hydrolase; *7* = F-box; *8* = Anchor protein; *9* = Hypothetical protein; *10* = SDR; *11* = Calmodulin-related protein; *12* = Hypothetical protein; *13* = Terpene synthase; *14* = Regulator of G protein signaling; *15* = Benzoic acid reductase-PKS; *16* = 3-Phosphoshikimate-1-carboxyvinyltransferase.

(1) *HfasTerp-94*: This biosynthetic gene cluster is located in contig 94 of the *H. fasciculare* genome. Its terpene synthase was predicted as a member of the 1,11 E,E-FPP carbon cyclization enzyme group. This group of enzymes is responsible for the production of *trans*-humulyl cation sesquiterpenes, such as humulene, caryophyllene, and protoilludane (Quin et al., 2014). When the sequences of these genes were BLAST searched against the *H. sublateritium* genome, an analogous biosynthetic gene cluster was found, with one exception—SDR3, which could not be found (Figures 3A–C).

(2) *HfasTerp-179*: This biosynthetic gene cluster was identified in contig 179 of *H. fasciculare*. This sesquiterpene synthase clade is identified as those based on germacradienyl derivatives. A BLAST search of the core enzyme and its adjacent biosynthetic genes against *H. sublateritium* genome indicated a matching cluster in *H. sublateritium* scaffold 11, but this appeared to be a larger cluster with two additional genes, likely beyond the end of the HfasTerp179 contig. Searching the Hfas genome with the two additional genes from the *H. sublateritium* scaffold 11 cluster identified these genes at the start of contig 615 of *H. fasciculare*, indicative of an incomplete genome assembly.
(3) *HfasTerp-804*: This cluster consisted of the terpene synthase that demonstrated high sequence similarity with the experimentally characterized oxidosqualene synthase located in scaffold 133 of *H. sublateritium*, a key enzyme for the production of the antitumor compound clavaric acid in *H. sublateritium* (Godio and Martín, 2009).
(4) *HfasTerp-255*: Manual sequence annotation of *H. sublateritium* scaffold 30 predicted three terpene synthases: HsubTerp-30a, HsubTerp-30b, and HsubTerp-30c. Phylogenetic analysis suggested HsubTerp-30a to function as squalene synthase and HsubTerp-30c as potential protoilludane synthase. BLAST searches of these genes against the *H. fasciculare* genome revealed high sequence similarity with several terpene synthases; the highest similarity (88%) was observed between HfasTerp-255 and HsubTerp-30c (Figure 4).

(5) *HfasTerp-147*: This BGC includes the only predicted terpene enzyme that follows the 1,10 3R neryl diphosphate (NPP) cyclization pattern. A homologous BGC was located in scaffold 11 of the *H. sublateritium* genome. Subsequent analysis of the tailoring genes of the *H. sublateritium* biosynthetic gene cluster suggested that the HfasTerp-147 gene cluster was assembled into two different contigs: HfasTerp-458 and HfasTerp-147 (Figure 5A).

(6) The *3,5-D* putative BGC: Among the shared hybrid biosynthetic gene clusters of *Hypholoma* spp., HfasTerp-104 and HsubTerp-38 appeared to have the necessary enzymes for the synthesis of the compound 3,5-D, such as 3-phosphoshikimate-1-carboxyvinyltransferase, benzoic acid reductase-polyketide synthase (PKS), short-chain dehydrogenase reductase (SDR), and glycoside hydrolase, highlighting their likely role in halogenated natural product synthesis (Figure 5B).

### Gene Clusters for Other Classes of Secondary Metabolites

**FIGURE 6:**
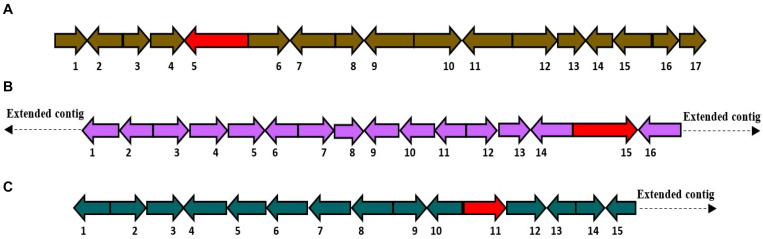
**(A)** Schematic of putative HfasPKS-221 (74 Kb) biosynthetic gene cluster. *1* = Triphosphate hydrolase; *2* = Multidrug resistance; *3* = Hypothetical protein; *4* = Alpha-beta hydrolase; *5* = PKS1; *6* = Zinc finger H2C2; *7* = Kinesin-like protein; *8* = Hypothetical protein; *9* = Actin binding protein; *10* = DNA binding protein; *11* = Acetylcholinesterase; *12* = Actin depolymerizing factor; *13* = Metalloproteases; *14* = Metalloproteases; *15* = Metalloproteases; *16* = *N*-acetylglucosaminyltransferase; *17* = Transcription factor. **(B)** Schematic of putative HfasNRPS-29 (76 Kb) biosynthetic gene cluster. *1* = mRNA-decapping enzyme; *2* = Peptidase aspartic; *3* = Glycoside hydrolase; *4* = Polyadenylate binding protein; *5* = Mitochondrial carrier protein; *6* = Ubiquitin protease; *7* = Acid phosphatase; *8* = AMP-dependent synthetase and ligase; *9* = Exocyst complex; *10* = DUF domain protein; *11* = Carbohydrate transporter; *12* = Adenine DNA glycosylase; *13* = Cargo transport protein; *14* = WD repeat-containing nucleolar rRNA; *15* = Oxidoreductase; *16* = NRPS; *17* = Acyl-CoA *N*-acyltransferase. A homologous biosynthetic gene cluster was found in scaffold 100 of the *Hypholoma sublateritium* genome. **(C)** Schematic of putative HfasSidA-14 (98 Kb) biosynthetic gene cluster. *1* = Transcription factor; *2* = Sterol desaturase; *3* = Cytochrome P450; *4* = Triphosphate hydrolase; *5* = Calcium transporting; *6* = Glycosyltransferase; *7* = GMC oxidoreductase; *8* = Amino acid transporter; *9* = Zinc binding protein; *10* = ABC transporter; *11* = Sid A; *12* = Glycoside hydrolase; *13* = Transporter; *14* = RNA-associated protein; *15* = F-box.

(7) *HfasPKS-221*: This biosynthetic gene cluster was positioned in contig 221 of *H. fasciculare*. Subsequent comparison with the *H. sublateritium* genome revealed an identical BGC situated in scaffold 53 (Figure 6A).

(8) *HfasNRPS-29*: This biosynthetic gene cluster was identified in contig 29 of *H. fasciculare*, and its ortholog cluster was found in scaffold 100 of the *H. sublateritium* genome (Figure 6B).
(9) *HfasSid-14*: This biosynthetic gene cluster was predicted in contig 14 of *H. fasciculare* and its ortholog located in scaffold 11 of *H. sublateritium* (Figure 6C).

### Silencing Experiments

**FIGURE 7:**
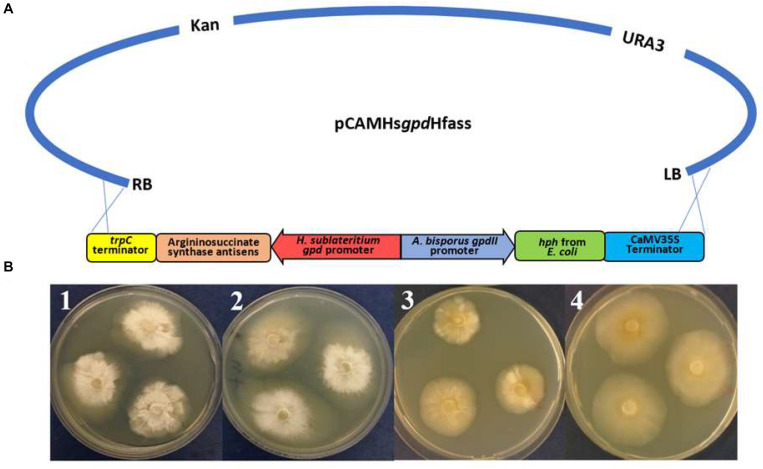
**(A)** Schematic representing the antisense vector pCAMHs*gpd*Hfas used for targeting argininosuccinate synthetase in *Hypholoma fasciculare*. **(B)** *H. fasciculare* wild type and antisense transformant 14 showing differences in the colony growth rate on potato dextrose agar (PDA) with and without arginine supplementation. *1* = *H. fasciculare* wild type on PDA medium; *2* = *H. fasciculare* wild type on PDA medium supplemented with 5 mM of arginine; *3* = *H. fasciculare* antisense transformant 49 on PDA medium; *4* = *H. fasciculare* antisense transformant 49 on PDA medium supplemented with 5 mM of arginine.

One method to validating gene function would be to silence the expression of each target gene and see whether there was a resulting depletion of a corresponding metabolite. Silencing was first assessed against the gene argininosuccinate synthetase. The silencing cassettes (Figure 7A) were transferred into *H. fasciculare* using *Agrobacterium*-mediated transformation following the protocol described in Al-Salihi et al. (2017). Silencing efficiency was assessed by comparing the hyphal growth pattern of the mutants with the wild type, with and without the external addition of arginine.

Several transformants showed a reduction in their colony size, among which transformant Hfas-asTR14 displayed the lowest growth rate (20 mm) compared to the wild type (30 mm), suggesting potential silencing in those lines. To further analyze this phenotype, replicates of transformant Hfas-asTR14 and the wild type (WT) were subcultured on potato dextrose agar (PDA) plates with and without 5 mM of the arginine supplement. Following 2 weeks of incubation at 25°C, the colony diameters were measured, and the results indicated potential successful gene silencing in transformant Hfas-asTR14, whereby silenced colonies demonstrated a slower rate of growth and different form of mycelia compared to the wild type (Figure 7B).

Following the argininosuccinate silencing experiments, terpene synthase silencing transformation was carried out in a similar manner. Classical selection on supplemented PDA plates was performed, and two selected silenced transformants for each line including two argininosuccinate synthetase transformants were further investigated for their biological activity using plate-based bioassay. Despite standardizing the size of the inoculum and culture conditions for all transformants, the silenced lines displayed variations in terms of colony diameter and inhibition zone. The observed morphogenesis of the silenced colonies was rigid, fluffy, wrinkled, condensed, and with loss of aerial mycelium (see Supplementary Figure 31).

Of nine silenced lines, HfasTerp-94bTR6 and HfasTerp-85bTR9 displayed the highest reduction in their zones of inhibition, 38 and 45%, respectively, while transformant HfasTerp-804TR8 and the argininosuccinate antisense Hfas-asTR49 produced larger inhibition zones (25%) compared to the wild type. Reductions from 12 to 32% were demonstrated by the remaining transformants (Table 2).

**TABLE 2:** Average colony and clearing zone diameters of two selected putative antisense transformants alongside the wild type.

**Colony on MEA plates**	**Average colony diameter (mm) ± SD of 3 technical replicates**	**Average colony diameter (mm) ± SD of 3 technical replicates**
HfWT	27 ± 0.7	32 ± 0.7
HfTerp94A-l	30 ± 1	26 ± 0.5
HfTerp94A-5	33 ± 0.7	24 ± 0.7
HfTerp94B-l	29 ± 0.5	24 ± 0
HfTerp94B-6	28 ± 0	20 ± 0.7
HfTerp85b-2	30 ± 1	22 ± 0
HfTerp85b-9	26 ± 1.4	18 ± 0.7
HfTerp 105-1	24 ± 1	24 ± 1.4
HfTerp 105-6	27 ± 0.5	22 ± 1.5
HfTerpl79-l	25 ± 0.7	28 ± 0.7
HfTerp 179-5	19 ± 0.7	26 ± 0.7
HfTerp342-6	32 ± 0.7	26 ± 1.5
HfTerp342-18	29 ± 1.4	28 ± 0.7
HfTerp804-2	21 ± 1	32 ± 0.7
HfTerp804-8	20 ± 0.5	40 ± 0.7
Hfas-as14	27 ± 0.7	34 ± 0.7
Hfas-as49	27 ± 0.7	40 ± 0.7

*MEA, malt extract agar; HfWT, wild type.*

### Gene Expression Analysis of *H. fasciculare* Silenced Lines

Genes HfasTerp-94a, HfasTerp-94b, and HfasTerp-105, *gpd*, and β-*tubulin* were used to detect their expression levels in the selected silenced transformants alongside the wild type. All transformants displayed reductions in their expression levels, attributed to the successful downregulation of their corresponding genes. Table 3 shows the fold differences of the selected genes over the conserved ones.

**TABLE 3:** RT-qPCR outcomes of the silenced lines.

**Sample**		**β-*tubulin* 2^−ΔCq^ average & ± SD**	***Gpd* 2^−ΔCq^ average & ± SD**
1	HfasTerp94aTRl	0.611 ± 0.085	0.031 ± 0
2	HfasTerp94aTR5	0.615 ± 0.191	0.004 ± 0.001
3	Hfas-WT94a	0.648 ± 0.068	0.002 ± 0.000
4	HfasTerp94bTR1	0.018 ± 0.003	0.002 ± 0.000
5	HfasTerp94bTR6	0.039 ± 0.006	0.001 ± 0.000
6	Hfas-WT94b	0.677 ± 0.067	0.002 ± 0.000
7	HfasTerpl05TR1	0.046 ± 0.043	0.001 ± 0.000
8	HfasTerpl05TR6	0.003 ± 0.000	0.000 ± 0
9	HfasWT 105	0.115 ± 0.008	0.000 ± 0.000

*Each gene was examined against two reference genes: β-tubulin and GAPDH.*

### Chemical Analysis of Silenced Lines

Different levels of SM production were observed among the silenced lines, particularly in transformants Hfas-asTR49, HfasTerp85bTR2, and HfasTerp85bTR9, where the production of a number of the molecules was reduced, including fascicularone G and naematoline. However, the production of the newly characterized (in *H. fasciculare*) 3,5-D showed no reduction in all transformants, indicating the involvement of a different type of key enzyme in its biological synthesis (Supplementary Figure 32 and Table 4).

**TABLE 4:** Growth pattern and mass detector response count per second of two selected putative antisense transformants for each silenced line alongside the wild type.

**Silenced line (4 mg/ml)**	**Predicted cyclization pattern**	**Growth pattern**	**Peak RT/predicted mass**		
			**Fas.G (A)**	**Naem (B)**	**3,5-D (C)**
			10.96/307-	11.70/353	13.10/218
*H. fasciculare*-WT		Ball-shaped mycelia	3.1 ×10^5^	2.9 × 10^5^	6.2 × 10^5^
HfTerp95A-1	1,11	=	9.7 × 10^5^	No mass	8.4 × l0^5^
HfTerp95A-5		=	6.1 ×10^3^	No mass	7.9 × 10^5^
HfTerp95B-1		=	7.6 × 10^5^	No mass	8.7 × 10^5^
HfTerp95B-6		Fine mycelia	5.4 ×10^5^	l×lO^4^	8.1 × 10^5^
HfTerp 105-1		Fine mycelia	8.2 × 10^5^	4.4 × 10^4^	1.9 × 10^5^
HfTerp 105-6		Fine mycelia	2.3 × 10^5^	3.7 × 10^4^	2.1 × 10^5^
HfTerp85b-2	?	Ball-shaped mycelia	1.2 × 10^5^	1.4 ×lO^4^	6.2 × 10^5^
HfTerp85b-9		Ball-shaped mycelia	2.2 × 10^5^	4.1 ×10^3^	5.5 × 10^5^
HfTerpl79-l	1,10	Fine mycelia	2 ×10^4^	1.5 × 10^4^	6.9 × 10^5^
HfTerp 179-5		Fine mycelia	1.9 × 10^5^	8.4 × 10^4^	3.5 × 10^5^
HfTerp342-6		Fine mycelia	1.1×lO^5^	2.6 × 10^4^	5 × 10^5^
HfTerp342-18		Ball-shaped mycelia	5.7 × 10^5^	6 ×10^5^	6.1 × 10^5^
HfTerp804-2	1,6	Fine mycelia	4.5 × 10^5^	2.3 × 10^4^	5.9 × 10^5^
HfTerp804-8		Ball-shaped mycelia	1.2 × 10^5^	2.1 ×10^3^	1.3 × 10^5^
Hfas-14		Fine mycelia	1.1×lO^5^	2.9 × 10^4^	4.7 × 10^5^
Hfas-49		Fine mycelia	2.5 × 10^5^	1.4 × 10^4^	5.8 ×10^5^

*WT, wild type; RT, retention time; Fas.G, fascicularone G; Naem., naematoline; 3,5-D, 3,5-dichloro-4-methoxybenzoic acid.*

### Heterologous Expression of Selected Sesquiterpene Synthases

**FIGURE 8:**
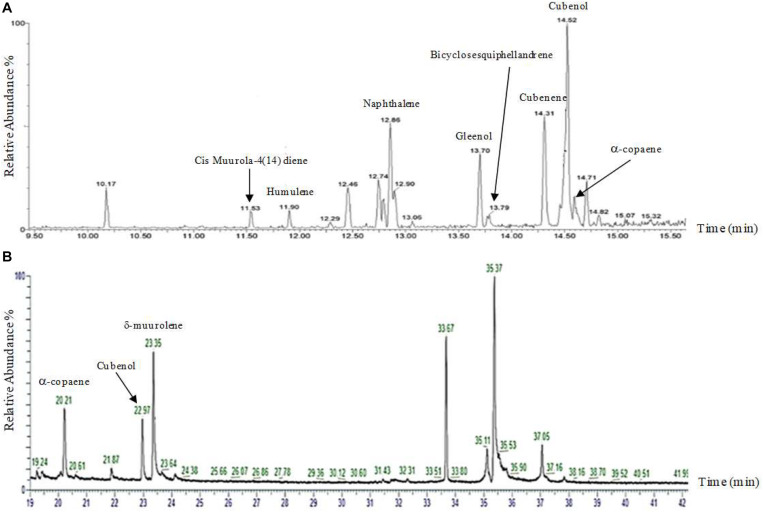
Gas chromatography–mass spectrometry (GC-MS) comparison of the sesquiterpene synthase Cop4. **(A)** GC-MS analysis of the NSAR1-Cop4 transformant. Eight compounds [muurola-4(14)diene, humulene, naphthalene, gleenol, cubenene, bicylosesquiphellandrene, Cubenol, and alpha-copaene] were characterized using HP-5 MS quartz capillary column (30 m × 0.25 mm, 0.25-μm film thickness). **(B)** GC-MS analysis of the NSAR1-Cop4 transformant. Three compounds (alpha-copaene, Cubenol, and sigma-muurolene) were characterized using HP-1 (50 m × 0.32 mm, 0.17-μm phase thickness).

Although silencing constructs has been proven successful for functional studies in *H. fasciculare*, its role in linking sesquiterpene metabolites to their specific biogenetic genes was inconclusive. We therefore adapted the vector pTYAGS-arg to express the selected sesquiterpene synthases in *A. oryzae* in order to further assess whether using *A. oryzae* as the expression host, as well as whether using different isolation techniques, would affect the measurement of the expression outcomes of some selected genes. *A. oryzae* transformants from a previous work were used (Al-Salihi et al., 2019), as well as six previously characterized fungal sesquiterpene synthases from two different basidiomycetes, *Omphalotus olearius* and *Coprinopsis cinerea*, as controls: cop-1, cop-2, cop-3, cop-4, omp-6, and omp-7 (Agger et al., 2009; Wawrzyn et al., 2012). Seven verified constructs—pTYGS-argHfasTerp179, pTYG-argCop-1, pTYG-argCop-2, pTYG-argCop-3, pTYG-argCop-4, pTYG-argOmp6, and pTYG-argOmp7—were independently transferred to *A. oryzae* strain NSAR1 protoplasts *via* polyethylene glycol (PEG)-mediated transformation reactions. Subsequent gas chromatography–mass spectrometry (GC-MS) analysis on previous (pTYGS-argHfasTerp94a, pTYGS-argHfasTerp94b, and pTYGS-argHfasTerp344) and current transformants confirmed our previous results of HfasTerp94a, HfasTerp94b, and HfasTerp344 (Al-Salihi et al., 2019) through the replication of humulene and caryophyllene production, although at lower levels. The production of most of the major products of the two controls, pTYG-argCop-3 and pTYG-argCop-4, were confirmed as well as the previously uncharacterized humulene in the Cop-3 extract (Figure 8 and Supplementary Figure 33). However, no product could be observed for the HfasTerp179 *A. oryzae* transformant.

### Co-expression and Chemical Analysis of Selected Biosynthetic Genes of the HfasTerp94 Gene Cluster

Adjacent genes (SDR1, SDR2, SDR3, and tyrosinase) of HfasTerp94 were selected for co-expression with NSAR1-humulene synthase. Due to the unsuccessful attempts of full-length cDNA amplification of the selected genes, an alternative approach of fragment amplification was chosen. *In silico* analysis predicted two exons for each SDR gene. Accordingly, four pairs of primers with 60 bp were used to amplify the two exons of each SDR from *H. fasciculare* genomic DNA (gDNA). A pTYGS-arg backbone was used for fragment recombination (Supplementary Figure 33). However, due to the prediction of several introns within the tyrosinase gene, a synthetic version was used.

**FIGURE 9:**
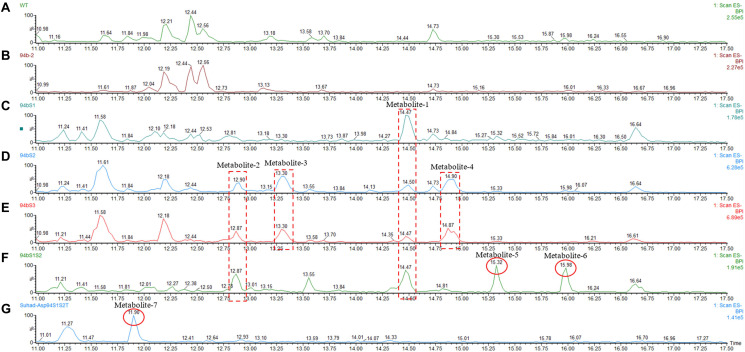
Negative ion LC-MS spectrum (-ESI) comparison of NSAR1-WT with transgenics of single, double, and triple biosynthetic genes of the humulene biosynthetic gene cluster. The genes investigated in this experiment were: one terpene cyclase (humulene synthase), three genes of short-chain dehydrogenase reductase (SDR), and one tyrosinase, all selected from the terpene synthase biosynthetic gene cluster located in contig 94 of the *Hypholoma fasciculare* genome. **(A)** NSAR1-WT. **(B)** NSAR1-humulene synthase. **(C)** NSAR1-humulene synthase-SDR1. **(D)** NSAR1-humulene synthase-SDR2. **(E)** NSAR1-humulene synthase-SDR3. **(F)** NSAR1-humulene synthase-SDR1-SDR2. **(G)** NSAR1-humulene synthase-SDR1-SDR2-Tyrosinase. The mass spectrum was selected from min 11 to min 17.50 to avoid peaks overlapping. In total, seven new peaks were detected at retention times (RTs) of 12.87, 13.30, 14.47, 14.90, 15.32, 15.98, and 11.90 within this comparison. The transgenic NSAR1-humulene synthase-SDR1 showed the lowest number of new peaks compared to the other transgenics: NSAR1-humulene synthase-SDR2, NSAR1-humulene synthase-SDR3, and NSAR1-humulene synthase-SDR1-SDR2.

**FIGURE 10:**
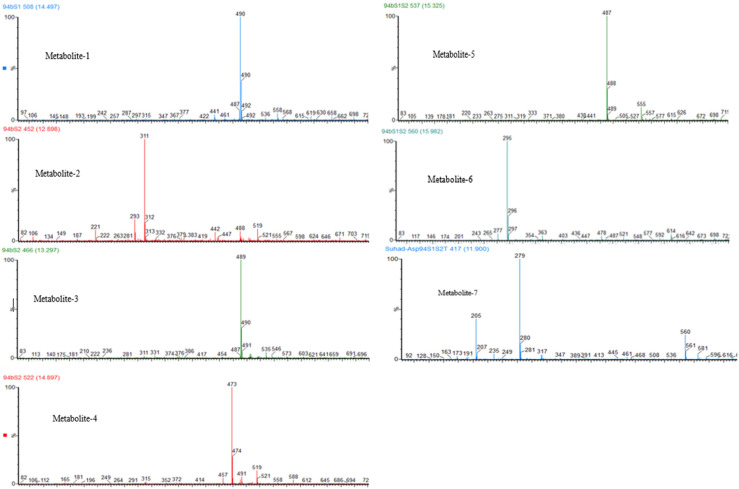
Negative ESI mass spectra of NSAR1 transgenics. Seven new metabolites were detected as formate adduct [M++HCO_2_] at: metabolite 1, *m*/*z* 490; metabolite 2, *m*/*z* 311; metabolite 3, *m*/*z* 489; metabolite 4, *m*/*z* 473; metabolite 5, *m*/*z* 487; metabolite 6, *m*/*z* 295; and metabolite 7, *m*/*z* 279.

Following successful transformation of the selected genes into *A. oryzae*, mass spectrum comparison between the five generated transformants (NSAR1-humulene synthase-SDR1, NSAR1-humulene synthase-SDR2, NSAR1-humulene synthase-SDR3, NSAR1-humulene synthase-SDR1-SDR2, and NSAR1-humulene synthase-SDR1-SDR2-Tyrosinase) and NSAR1-humulene synthase, crude extracts were evaluated; in total, seven new peaks were produced. Since the analysis was performed in electrospray ionization (ESI) negative mode, it was assumed that the observed *m*/*z* values would be 46 mass units higher due to the formation of a formic acid adduct. For the humulene synthase-SDR1 transgenic, one new peak (metabolite 1) was observed at 14.47 min, with an *m*/*z* of 489. In addition to metabolite 1, the chromatograms of both NSAR1-humulene synthase-SDR2 and NSAR1-humulene synthase-SDR3 produced three new peaks, eluting at 12.90 min (metabolite 2), 13.30 min (metabolite 3), and 14.90 min (metabolite 4), with *m*/*z* 487, 489, and 473, respectively. Although the chemical profile of the transgenic NSAR1 with humulene synthase-SDR1-SDR2 showed no accumulation for both metabolites 3 and 4, new peaks at 15.32 and 15.98 min with *m/z* 487 and 295, respectively, were observed. The fragmentation patterns of metabolites 1 and 2 were identical to metabolites 3 and 5, respectively, indicative that they may be isomeric compounds (Figures 9, 10).

### Genome Accession Number

The genome sequences of *H. fasciculare* can be freely accessed on the NCBI platform under the accession number JAEKIV010000000.

## Discussion

The continued rise in antibiotic resistance has prompted scientists to look for new ways to discover potential new drugs. While mushroom-forming fungi have been reported as a rich repository of structurally diverse natural products, the limited access to their metabolomic and genomic data poses a bottleneck to the development of their potential. In this study, the prospective of selected fungi as a source of novel antibiotics is investigated. Eight of the nine species selected displayed antimicrobial properties, of which *Hypholoma* showed a notable range of antimicrobial activity. This is reflected in its metabolic variety and the flexibility in their response to various competitive ecological niches (Boddy and Watkinson, 1995), which could be potentially important in defeating resistant microbes.

Direct bioautography and subsequent chromatographic, mass spectral, and NMR spectroscopic analysis enabled us to unveil the antibacterial potency of an aromatic compound, 3,5-D. This molecule was first characterized from *Stropharia* sp. through the observation by Thines et al. (1995) of two aromatic chemical shifts (aromatic proton and the methoxy group) and the carbon they are attached to at 130.4 and 155.8 ppm, respectively. Here, we fruitfully used both 1D and 2D spectroscopic analyses to provide solid proof of the presence of the other substituent groups (Cl, CH_3_, and COOH) and to allow the first elucidation of 3,5-D in *Hypholoma* genus and second within fungi kingdom. Previous studies have reported that chlorinated compounds are synthesized through the shikimate pathway (Jensen et al., 1994; Mester et al., 1997; Hage et al., 1999). This pathway involves the conversion of the phenylalanine precursor to benzoic acid derivatives *via* sequential condensation, hydroxylation, and chlorination. Interestingly, the predicted HfasTerp104 gene cluster includes enzymes that are likely responsible for the synthesis of 3,5-D, including the benzoic acid reductase-PKS, SDR, glycoside hydrolase, and the multifunctional 3-phosphoshikimate-1-carboxyvinyltransferase. 3-Phosphoshikimate-1-carboxyvinyltransferase is a multidomain enzyme with a main role in catalyzing the conversion of phenylalanine-like compounds to cinnamic acid derivatives. Subsequent hydroxylation and reduction on the resulting acids lead to the production of anisaldehyde isomers such as 3,5-D (Field et al., 1996). The biological activity of chlorinated natural products is well documented (Hautzel and Anke, 1990; Pfefferle et al., 1990; Becker et al., 1994). Identifying their BGCs is a step forward in the biochemical manipulation of such understudied potential potent antimicrobial agents.

Unlike *H. sublateritium*, the *H. fasciculare* genome was assembled into greater number of short contigs. It is likely that our assembly method dispersed the resulted gene clusters of *H. fasciculare* into several short contigs rather than one long scaffold, as the case of its related *H. sublateritium* genome assembled by JGI. Nevertheless, phylogenetic comparisons and in-depth manual curation with the *H. sublateritium* genome sequences guided the annotation of a set of hidden biosynthetic gene clusters in both species. Most of the 15 putative gene clusters contain key genes encoding terpene, PKS, non-ribosomal peptide synthase (NRPS), Sid, and their related modifying genes, which represent the main features of fungal SM biosynthetic pathways. The *in silico* analysis suggests that the predicted terpene clusters are likely responsible for the production of terpene-like compounds, particularly sesquiterpenes and triterpenes (previously reported compounds in *Hypholoma* spp.). A putative siderophore biosynthetic pathway and a multimodule NRPS core enzyme were also found in the *H. fasciculare* genome, and subsequent manual curation led to the identification of a putative biosynthetic cluster in both *Hypholoma* species. Most often, multidomain NRPSs are involved in the synthesis of SM with medicinal properties (Sayari et al., 2019), indicative that the *Hypholoma* species may produce NRPSs and siderophore-based metabolites that require further investigation. Several hybrid clusters containing two different cyclase enzymes (e.g., a predicted PKS and a terpene synthase) were also distinguished. Such biosynthetic clusters often coordinate the production of meroterpenoid molecules such as melleolide (Engels et al., 2011), as well as the newly characterized 3,5-D. Further annotation of the clusters suggested that the PKS of Hfasterp104 is actually a benzoic acid reductase that produces benzoate precursors. This enzyme, along with the multifunctional gene 3-phosphoshikimate-1-carboxyvinyltransferase, catalyzes five sequential steps in the shikimate pathway and is the key biosynthetic producer of phenolic compounds in plants (Santos-Sánchez et al., 2019).

Gene silencing was performed to downregulate the expressions of some selected terpene synthetase genes (alongside argininosuccinate synthetase), demonstrating that RNA silencing is an effective tool in knocking down transcripts of argininosuccinate. The silenced transformants showed variability in their phenotypes, represented by the slow growth rates or the production of unusual forms of mycelia. These phenotypic variations are congruent with other published silencing works in basidiomycetes (Eastwood et al., 2008; Costa et al., 2009). However, extended silencing work on sesquiterpene synthases reveals a crossed role of such enzymes in the synthesis of the *H. fasciculare* SMs, where the production of several SMs was prevented better as a result of silencing one enzyme. For example, silencing HfasTerp84b resulted in the absence of most metabolites that were observed in the chemical profile of the WT. This promiscuous feature of basidiomycetes sesquiterpene synthase has been reported previously (Agger et al., 2009; Wawrzyn et al., 2012), where independent heterologous expressions of a group of sesquiterpene synthases from two different basidiomycetes, *O. olearius* and *C. cinerea*, in *E. coli* resulted in several chemically distinguishable sesquiterpene molecules for each enzyme. Interestingly, the GC-MS analysis of *A. oryzae* transgenics of some of the enzymes used in Schmidt’s work (Agger et al., 2009; Wawrzyn et al., 2012) confirmed the fact that *A. oryzae* is a feasible tool in linking SM to their specific enzymes and that its metabolomic system has no effect on the final product of sesquiterpene core enzymes, as most major products of Schmidt’s group were replicated here. The absence of the remaining compounds from the observation in the other *Aspergillus* transgenics is likely due to the use of a different isolation method, as the detection of compounds is influenced by the isolation method and the chromatographic system used during the characterization process (Jeleń, 2003).

The detection of humulene in the GC-MS analysis of HfasTerp94 motivated the further expression of its related biosynthetic genes in *A. oryzae*. In this cluster, three SDR genes were predicted alongside other distinctive genes including zinc carboxypeptidase and tyrosinase. SDR genes belong to a large family of oxidoreductases that have a key role in oxidoreductase reactions through synthesizing the oxidation of metabolites (Kallberg et al., 2010). Co-expression of SDR genes with the humulene synthase was therefore hypothesized to result in the production of oxidized humulene derivatives. Different combinations of genes (dual and triple gene vectors) were therefore transformed into NSAR1 to potentially reveal the function of each enzyme. The crude chemical extract of the positive *Aspergillus* transformants were then analyzed *via* GC-MS, leading to the detection of several new peaks. This suggested that the SDR and tyrosinase protein sequences were correctly annotated and that they can further modify the humulene backbone. Based on previously reported results, humulene can be directly deprotonated to produce sesquiterpene by-products (Agger et al., 2009). Although the observation of two new metabolites (5 and 6) in the chemical profile of NSAR1-SDR1-SDR2-Tyrosinase may indicate the potential conversion of metabolites 3 and 4 of NSAR1-SDR1-SDR2, further evidence of the compound’s identity (such as a full NMR characterization) is needed to precisely link the new SMs to the corresponding genes. It is also worth noting that the expressions of some modifying enzymes (e.g., cytochrome P450 and SDR) may impact the metabolic pathway of the utilized host, *A. oryzae* (Shinohara et al., 2016), and that the compounds observed are the result of combinatorial efforts between the inserted genes and the native genes of the host. This hypothesis, however, needs further laboratory-based evidence.

## Conclusion

Mushroom-forming fungi offer the potential for the production of new molecules with novel biological activities that have the potential to be medicinally useful, especially in the development of new antibiotics. However, only a small number of these fungi has been fully explored at both the genetic and chemical levels. To address problems associated with SM production in *H. fasciculare* (low yield and difficulties in growth), an in-depth sequence analysis of the *H. fasciculare* genome was performed. This led to the characterization of several putative biosynthetic gene clusters that are likely to be involved in the production of the previously reported compounds in this fungus. When we experimentally tested our *in silico* genome prediction *via* RNAi-mediated and heterologous expression in *A. oryzae*, we further demonstrated that RNAi silencing is still an efficient tool in triggering the downregulation of gene expression and could be deployed in other genetic manipulations as in *H. fasciculare* and that sesquiterpene synthases are promiscuous enzymes. The study also permitted the chemical characterization of a biologically active meroterpenoid (3,5-D) as well as the *in silico* prediction of the biosynthetic gene cluster associated with it. Prominently, this cumulative knowledge of the bioactive molecules’ structures and their biosynthesis and pharmacokinetic properties will help in developing a molecular framework to prolifically employ this fungus in the battle against antimicrobial resistance.

## Data Availability Statement

The raw data supporting the conclusions of this article will be made available by the authors, without undue reservation.

## Author Contributions

SA-S designed and performed the experiments, analyzed data, and wrote the manuscript. IB provided some GC-MS samples analysis. RA-S and KS participated in solving the chemical structure of 3,5-D compound. PG participated in 3,5-D HPLC run and provided feedback on its fragmentation. AB and GF devised and supervised the project. All authors contributed to the article and approved the submitted version.

## Conflict of Interest

The authors declare that the research was conducted in the absence of any commercial or financial relationships that could be construed as a potential conflict of interest.
