# Supplementary material for: Further Biochemical Profiling of Hypholoma fasciculare Metabolome Reveals Its Chemogenetic Diversity
Source: Front Bioeng Biotechnol. 2021 May 24;9:567384. doi: 10.3389/fbioe.2021.567384 (PMC8181146; doi:10.3389/fbioe.2021.567384)
Supplement: Supplementary file 1 [file Data_Sheet_1.PDF]

Further biochemical profiling of *Hypholoma fasciculare* metabolome reveals its chemo-genetic diversity

Suhad A.A. Al-Salihi[1§*], Ian D. Bull[2], Raghad Al-Salhi[3], Paul J. Gates[2], Kifah Salih[4], Andy M. Bailey[1*], Gary D. Foster[1*]

1 School of Biological Sciences, University of Bristol, 24 Tyndall Avenue Bristol, BS8 1TQ, UK.
2 School of Chemistry, University of Bristol, Cantock's Close, Bristol, BS8 1TS, UK.
3 Chemistry Department, University of Mustansiriyah, Baghdad, 10052, IRAQ.
4 Chemistry and Earth Sciences, Qatar University, Doha, Qatar.
§ Present address: Applied Science Department, University of Technology, Baghdad, 10066, IRAQ.
[*]To whom correspondence should be addressed: Gary Foster Gary.Foster@bristol.ac.uk, Andy Bailey Andy.Bailey@bristol.ac.uk and Suhad Al-Salihi suhadbiotechnology@gmail.com

Supplementary information

Figure S1: Represents characterised compounds from *H. fasciculare*.

Table S1: Selected experimentally characterised terpene cyclase, polyketide synthase, non-ribosomal peptides and their sequences details used in Hypholoma genome comparison.

Table S2: Predicted SM of Hypholoma spp. using antiSMASH and Local BLAST

Figure S2 Bioassay test to evaluate the antimicrobial activity of *Paxillus involutus*.

Figure S3: Bioassay test to evaluate the antimicrobial activity of *Coniophora puteana.*

Figure S4: Bioassay test to evaluate the antimicrobial activity of *Fomitiporia mediterranea.*

Figure S5: Bioassay test to evaluate the antimicrobial activity of *Postia placenta*.

Figure S6: Bioassay test to evaluate the antimicrobial activity of *Gloeophyllum trabeum*.

Figure S7: Bioassay test to evaluate the antimicrobial activity of *Laccaria bicolor.*

Figure S8: Bioassay test to evaluate the antimicrobial activity of *Suillus luteus.*

Figure S9: TLC plate developed in semi polar system and visualized under UV 320nm showing separated compounds of *H. fasciculare*.

Figure S10: TLC plate developed in non-polar system and visualized under UV 320nm showing separated compounds of *H. fasciculare*.

Figure S11: TLC plate developed in semi polar system and visualized under UV 320nm showing separated compounds of *H. sublateritium*.

Figure S12: TLC plate developed in non-polar system and visualized under UV 320nm showing separated compounds of *H. sublateritium*.

Figure S13: Direct bioautography of *H. fasciculare* crude extracts showing the antimicrobial activity of different crude extracts developed in polar solvents.

Figure S14: Direct bioautography of *H. fasciculare* crude extracts showing the antimicrobial activity of different crude extracts developed in semi polar solvents.

Figure S15: Direct bioautography of *H. fasciculare* crude extracts showing the antimicrobial activity of different crude extracts developed in non-polar solvents.

Figure S16: LC-MS chromatograms electrospray mass negative mode (ES-).

Figure S17: ^{1}H NMR (125 MHz) spectrum of 3, 5-dichloro-4-methozybenzoic acid.

Figure S18: ^{13}C NMR (500 MHz) spectrum of 3, 5-dichloro-4-methozybenzoic acid.

Figure S19: COSY (500 MHz) spectrum of 3, 5-dichloro-4-methozybenzoic acid.

Figure S20: HSQC (500 MHz) spectrum of 3, 5-dichloro-4-methozybenzoic acid.

Figure S21: HMBC (500 MHz) spectrum of 3, 5-dichloro-4-methozybenzoic acid.

Figure S22: Predicted gene cluster of Hfas-terp105 and the homologous cluster (Hsub-terp83) in *H. sublateritium*

Figure S23: Predicted gene cluster of *H. sublateritium* terpene synthases (30, 113, 38) and their homologous in *H. fasciculare*.

Figure S24: Predicted gene cluster of *H. sublateritium* terpene synthases (11A, 205, 28) and their homologous in *H. fasciculare.*

Figure S25: Predicted gene cluster of *H. sublateritium* terpene synthases (133, 167) and their homologous in *H. fasciculare*.

Figure S26: Predicted gene cluster of *H. sublateritium* terpene synthase 11B and the homologous cluster in *H. fasciculare*

Figure S27: Predicted gene cluster of *H. sublateritium* terpene synthase 99 and the homologous cluster in *H. fasciculare*.

Figure S28: Predicted gene cluster of *H. fasciculare* and *H. sublateritium* PKS.

Figure S29: Predicted gene cluster of *H. fasciculare* and *H. sublateritium* NRPS.

Figure S30: Predicted gene cluster of *H. sublateritium* SidA and the homologous cluster in *H. fasciculare*.

Figure S31: Represents randomly selected mutants of *H. fasciculare* terpene synthase gene silencing experiments along with two GFP transformants and the wild type.

Figure S32: Diode array chromatograms for *H. fasciculare* WT and putative silenced transformants crude extracts.

Figure S33: GC-MS spectrum comparison of A. Sesquiterpene synthase Cop3 and B. Humulene standard.

Figure S34: Schematic representation of the principle of constructing the pTYGS-arg-SDR plasmid.

Table S3. List of primers used for *H. fasciculare* terpene synthase antisense plasmids construction.

Materials and Methods.
Bioassay preparation.

References.

A

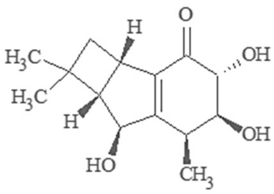

$C_{14}H_{20}O_4$ (252.31)

Fascicularone A

(sesquiterpenoids)

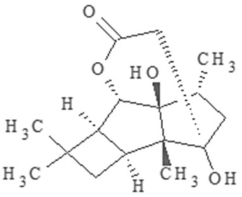

$C_{15}H_{22}O_4$ (266.337)

Fascicularone B

(sesquiterpenoids)

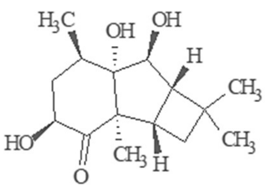

$C_{15}H_{24}O_4$ (268.353)

Fascicularone C

(sesquiterpenoids)

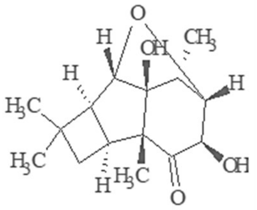

$C_{15}H_{22}O_4$ (266.337)

Fascicularone D

(sesquiterpenoids)

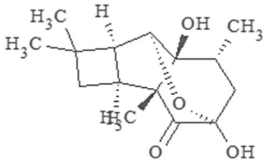

$C_{15}H_{22}O_4$ (266.337)

Fascicularone E

(sesquiterpenoids)

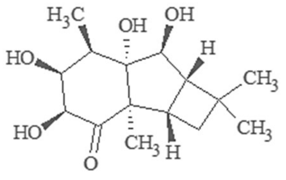

$C_{15}H_{24}O_5$ (284.353)

Fascicularone F

(sesquiterpenoids)

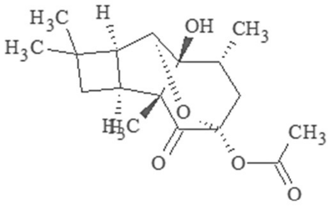

$C_{17}H_{24}O_5$ (308.375)

Fascicularone G

(sesquiterpenoids)

References: Shiono *et al.*, 2004a and b; Shiono *et al.*, 2005.

B

$C_{36}H_{60}O_8$ (620.868)

Fasciculic acid A

(triterpenoid)

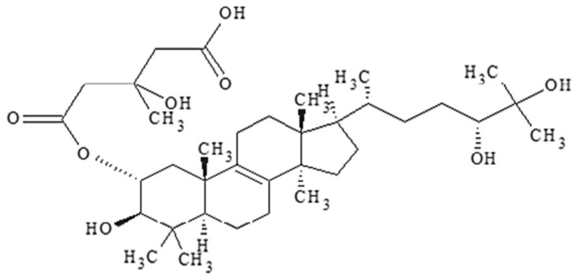

$C_{36}H_{60}O_9$ (636.867)

Fasciculic acid B

(triterpenoid)

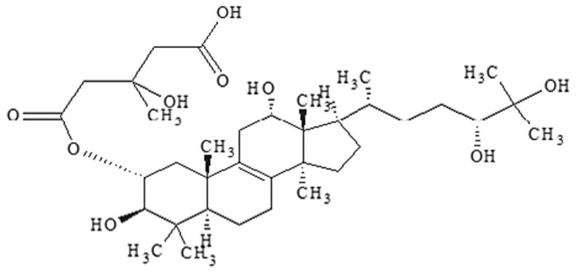

$C_{38}H_{63}NO_{11}$

(709.918)

Fasciculic acid C

(triterpenoid)

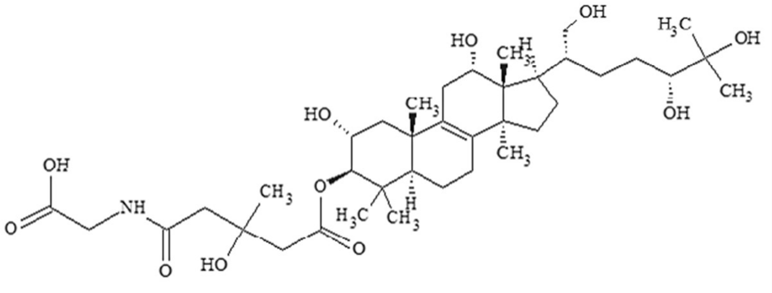

References: Takahashi *et al.*, 1989

C

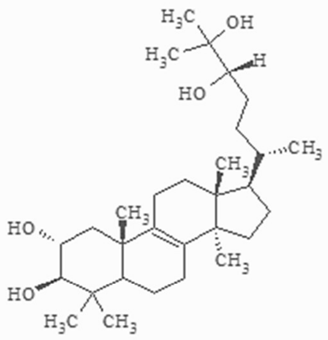

$C_{30}H_{52}O_4$ (476.74)

Fasciculol A

(triterpenoid)

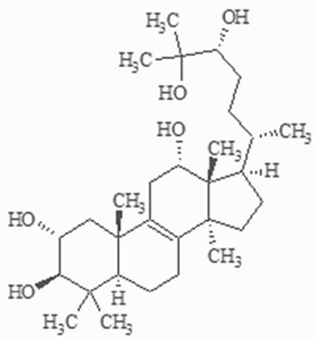

$C_{30}H_{52}O_5$ (492.74)

Fasciculol B

(triterpenoid)

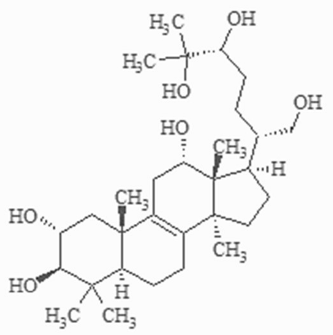

$C_{30}H_{52}O_6$ (508.739)

Fasciculol C

(triterpenoid)

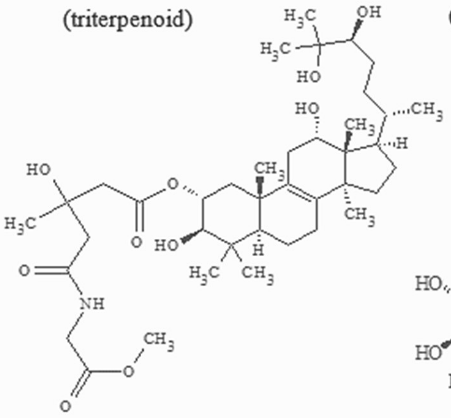

$C_{39}H_{65}NO_{10}$ (707.946)

Fasciculol D

(triterpenoid)

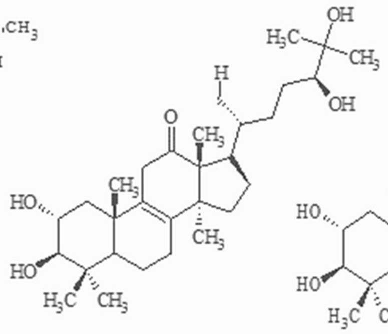

$C_{30}H_{50}O_5$ (490.3658)

Fasciculol H

(triterpenoid)

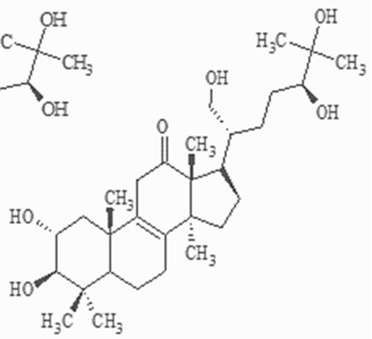

$C_{30}H_{50}O_6$ (506.3607)

Fasciculol I

(triterpenoid)

References : Kubo *et al.*, 1985; Shi *et al.*, 2011.

D

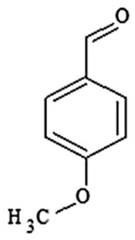

$C_8H_8O_2$ (136.0524)

p- anisaldehyde

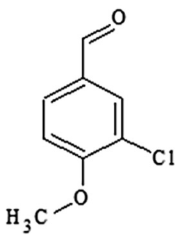

$C_8H_7ClO_2$ (170.595)

3-chloro-*p*-anisaldehyde

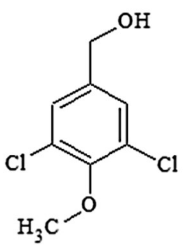

$C_8H_8Cl_2O_2$ (205.9901)

3,5 dichloro-4 methoxybenzyl alcohol

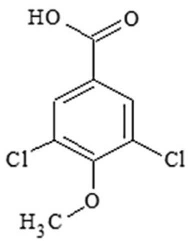

$C_8H_6Cl_2O_3$ (219.9694)

3,5-dichloro-4-methoxybenzoic Acid

References : Aqueveque et al., 2006 ; Al-Salihi et al., current study.

E

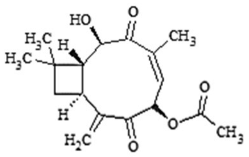

$C_{17}H_{22}O_5$ (306.359)

Naematolon

(sesquiterpenes)

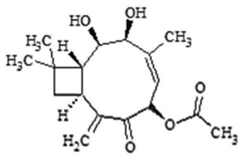

$C_{17}H_{24}O_5$ (308.375)

Naematolin

(sesquiterpenes)

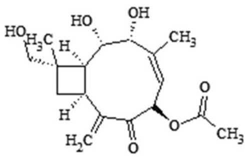

$C_{17}H_{24}O_6$ (324.374)

Naematolin B

(sesquiterpenes)

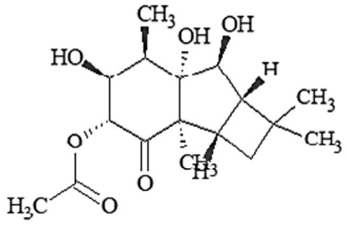

$C_{17}H_{26}O_6$ (326.39)

Naematolin C (sesquiterpenes)

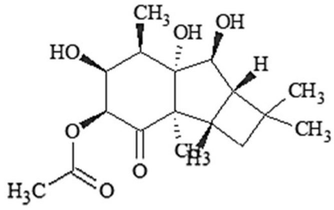

$C_{17}H_{26}O_6$ (326.39)

Naematolin G

References : Ito *et al*., 1967; Backens, *et al*., 1984 ; Doi *et al*., 1986 ; Doi *et al*., 1990.

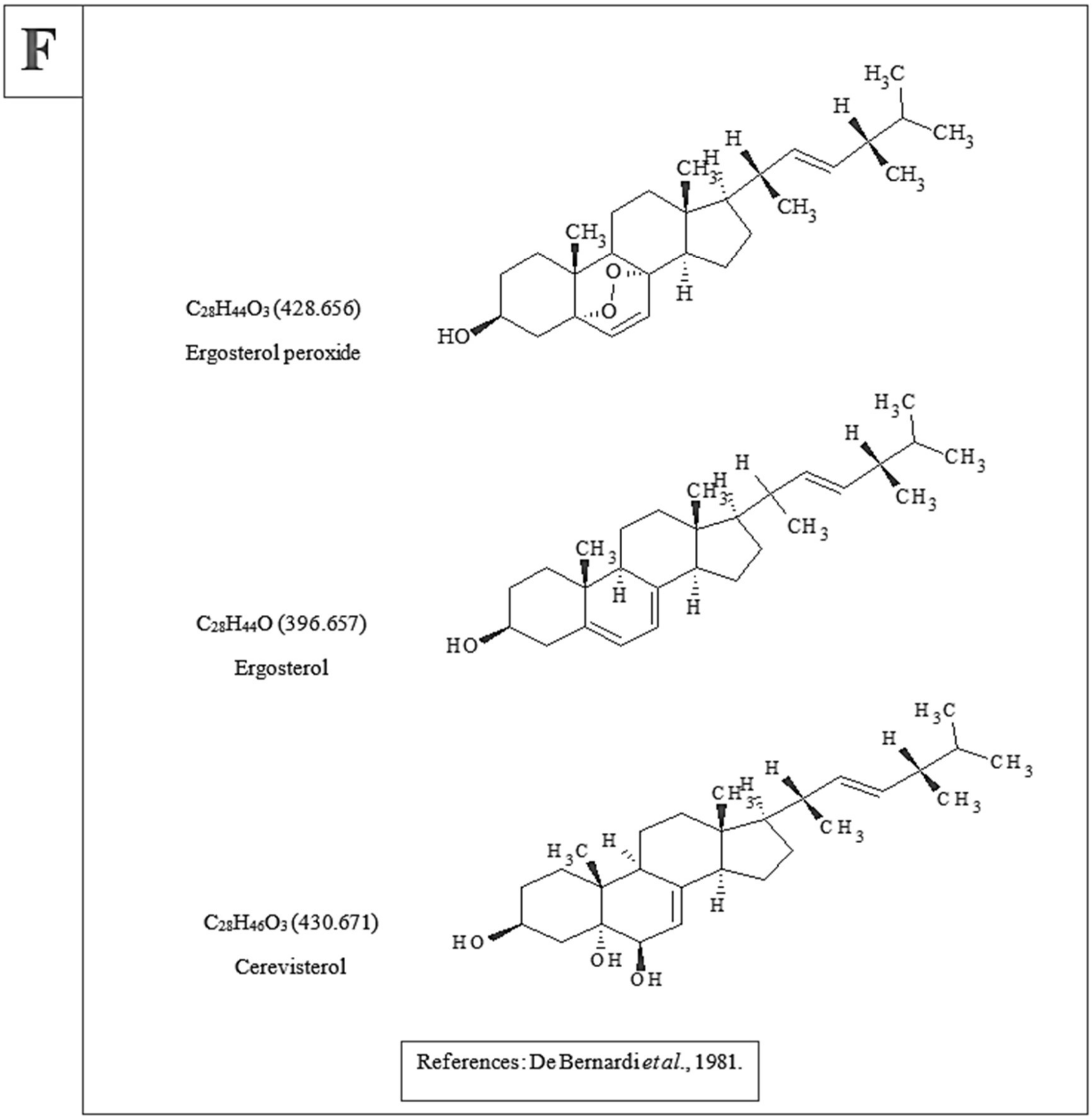

Figure S1: Represents characterised compounds from *H. fasciculare,* covers 1967-2019. Molecular weight and chemical formula were illustrated in this table.

A- Fascicularone isomers, B- Fasciculic acid isomers, C- Fasciculol isomers, D- *p*- anisaldehyde isomers, E- Naematolin isomers and F- Ergosterol isomers

Table S1: Selected experimentally characterised terpene cyclase, polyketide synthase, non-ribosomal peptides and their sequences details used in *Hypholoma* genome comparison.

SM type		SM cyclase or synthetase	Enzyme feature	NCBI or JGI protein ID	Organism	References
**1**	TC	Ompol1| (1)	1,10 cyclization of E, E, F-PP	1311	Omphalotus olearius	Wawrzyn et al., 2012
		Copci1| (3)		14587	Coprinus cinereus	Agger et al., 2009
		Stehi1	1,10 cyclization of 3R-NPP	128017	Stereum hirsutum	Quin et al., 2013
		Ompol1| (4)		1447	Omphalotus olearius	Wawrzyn et al., 2012
		Stehi1|	1,11 cyclization of E,E,FPP	64702	Stereum hirsutum	Quin et al., 2013
		Ompol1| (7)		2271	Omphalotus olearius	Wawrzyn et al., 2012
		Ompol1| (9)	1,6 cyclization of 3R/S-NPP	3258	Omphalotus olearius	Wawrzyn et al., 2012
		Copci1| (6)		20268	Coprinus cinereus	Quin et al., 2013
**2**	PKS	LDKS	Lovastatin diketide synthase	Q9Y7D5	*Aspergillus terreus*	Meehan et al., 2010
		PKSA	Sterigmatocystin polyketide synthase	Q12053.1	*Aspergillus parasiticus*	Feng and Leonard, 1995
		hybrid PKS-NRPs	Beta-Keto acyl synthase	Q8J0F7	*Penicillium citrinum*	Abe et al., 2002
		MSAS	6-Methylsalicyclic acid synthase	AAC49814.1	*Aspergillus terreus*	Fujii et al., 1996
		ArmB		JQ801748	Armillaria mellea	
		PKS1		169950	Coniophora puteana	
		PKS1		116317	Gloeophyllum trabeum	
**3**	NRPS	AMT	AM-toxin synthetase	AAF01762.1	*Alternaria alternata*	Johnson et al., 2000
		SidD	SidD	748662.1	*Aspergillus fumigatus*	Nierman et al., 2005

Table S2: Predicted SM of *Hypholoma spp*. using antiSMASH and Local BLAST

Cluster type	Predicted SM gene clusters using antiSMASH	
	H. fasciculare	*H. sublateritium*
**Terpene**	Contigs: 70, 85, 94, 105, 173, 179	Scaffolds: 11a, 30, 43, 133
**Siderophore**	Contig 14	Scaffold 11
**NRPS**	Contig 43	Scaffold 7
**T1pks**	Contig 221	Scaffold: 12, 53
**Predicted SM gene clusters using Local BLAST**		
**Terpene**	Contigs: 10, 147, 266, 339, 378, 28	Scaffolds: 11b, 28, 38, 81, 113, 116, 167, 205
**PKS**	Hfas85	Hsub116
**NRPS**	Hfas29	Hsub99

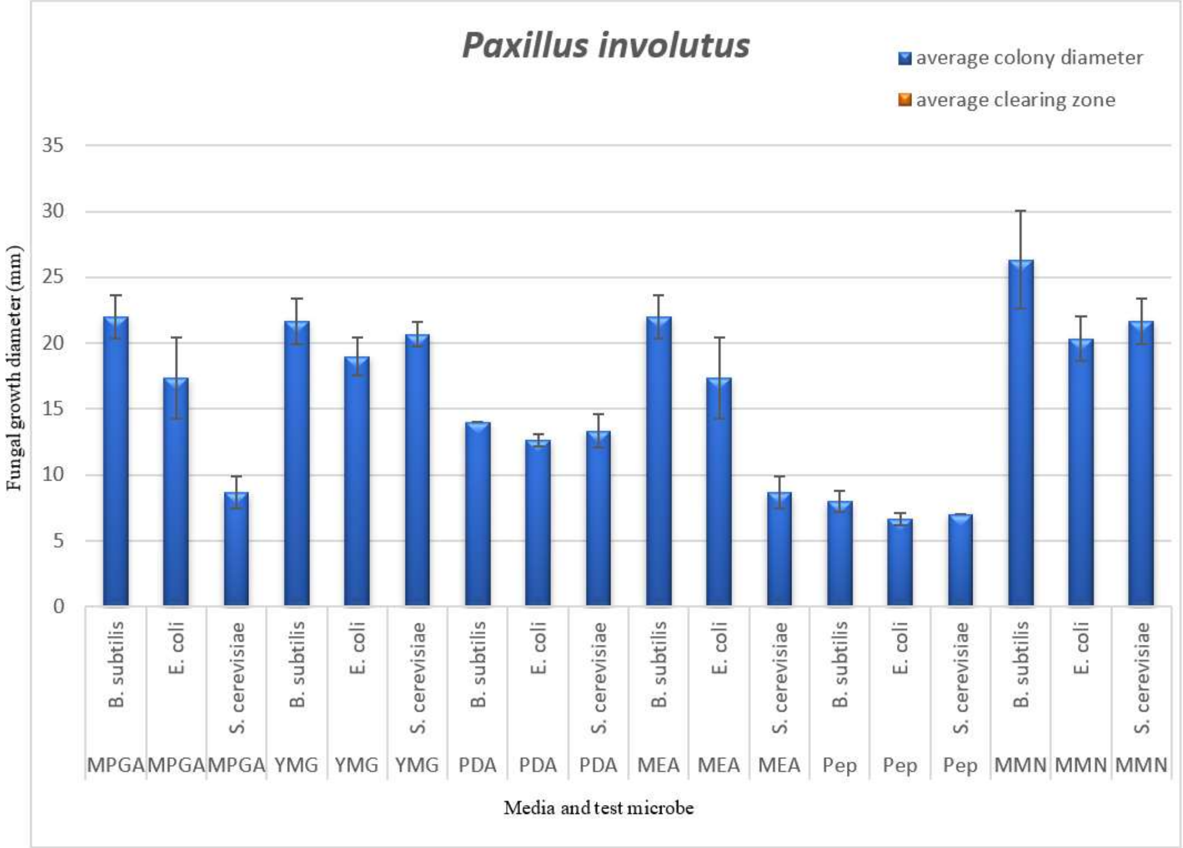

Figure S2: Bioassay test to evaluate the antimicrobial activity of *P. involutus* growing on different media against *B. subtilis*, *E. coli* and *S. cerevisiae*. MPGA = malt peptone glucose agar, YMG = yeast extract malt, PDA = potato dextrose agar, MEA = malt extract agar, Pep = peptone agar, MMN = minimum melin norkans agar. Error bars indicates the standard deviation of three technical replicates measurement for both fungal colony diameter (column in blue) and inhibition zone diameter (column in red).

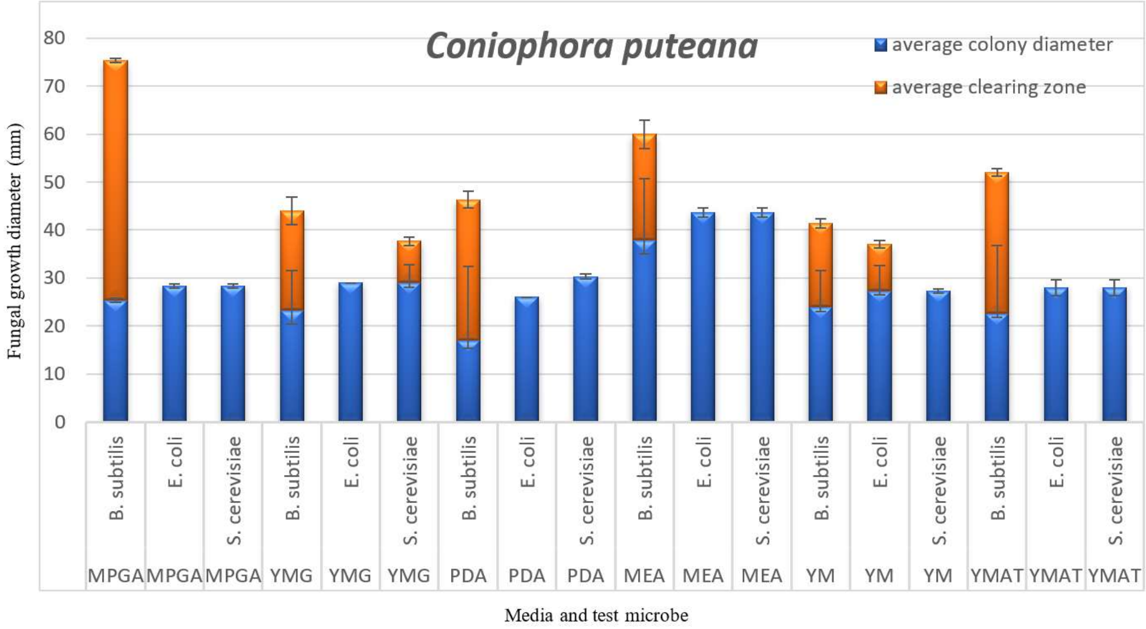

Figure S3: Bioassay test to evaluate the antimicrobial activity of *C. puteana* growing on different media against *B. subtilis*, *E. coli* and *S. cerevisiae*. MPGA = malt peptone glucose agar, YMG = yeast extract malt, PDA = potato dextrose agar, MEA = malt extract agar, Pep = peptone agar, YM = yeast malt extract, YMAT = yeast malt dextrose peptone agar. Error bars indicates the standard deviation of three technical replicates measurement for both fungal colony diameter (column in blue) and inhibition zone diameter (column in red).

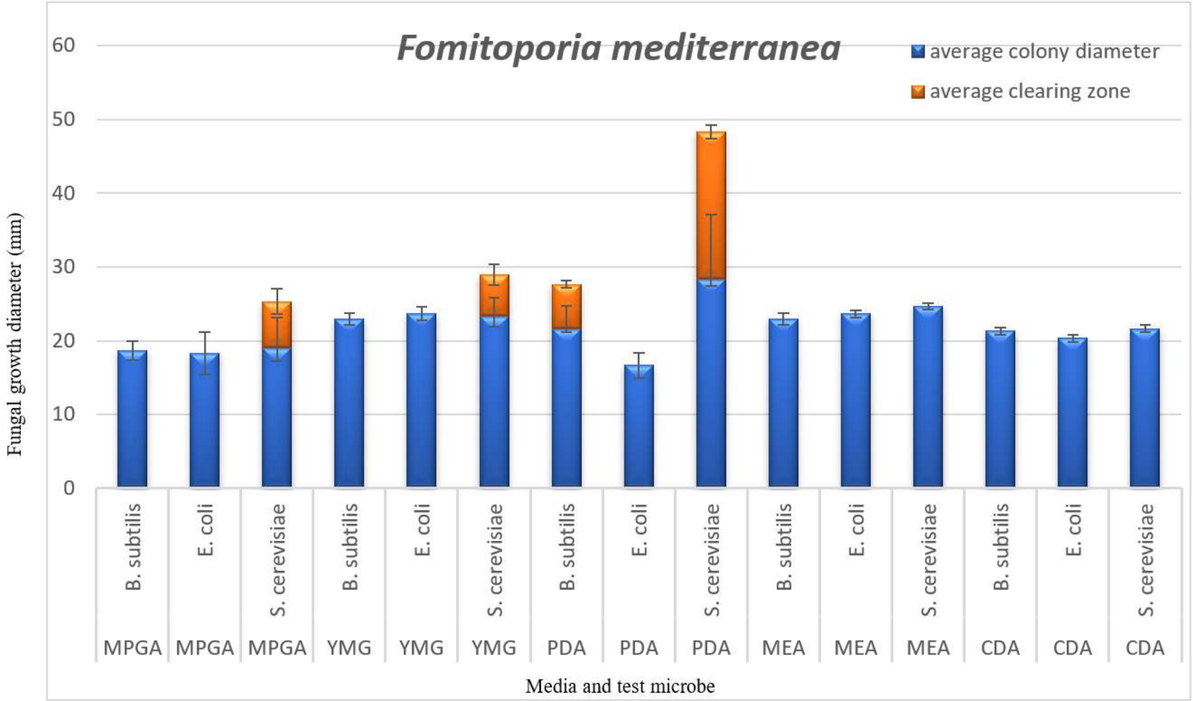

Figure S4: Bioassay test to evaluate the antimicrobial activity of *F. mediterranea* growing on different media against *B. subtilis*, *E. coli* and *S. cerevisiae*. MPGA = malt peptone glucose agar, YMG = yeast extract malt, PDA = potato dextrose agar, MEA = malt extract agar, CDA = Czapek Dox agar. Error bars indicates the standard deviation of three technical replicates measurement for both fungal colony diameter (column in blue) and inhibition zone diameter (column in red).

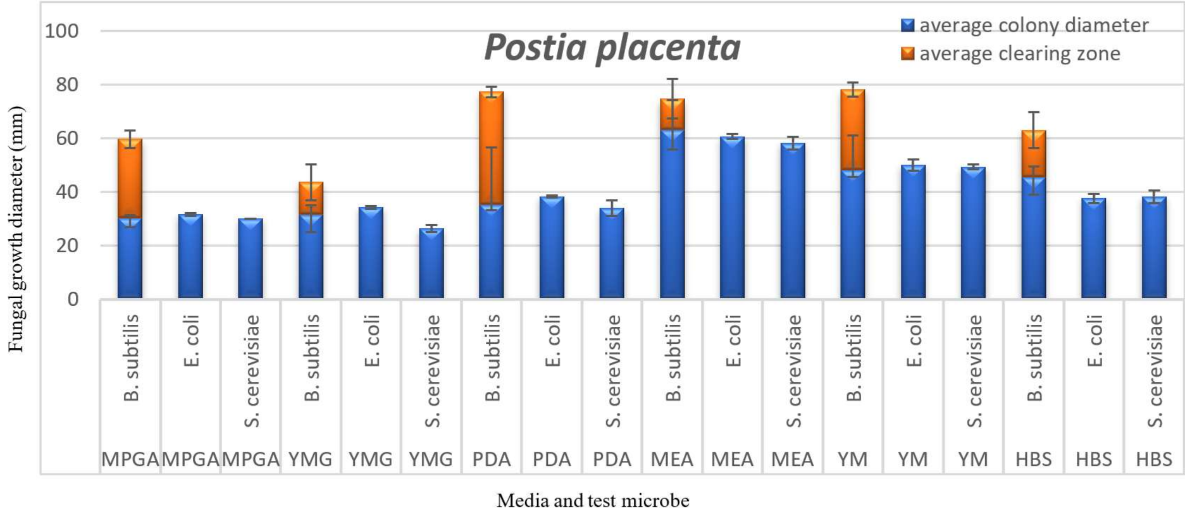

Figure S5: Bioassay test to evaluate the antimicrobial activity of *P. placenta* growing on different media against *B. subtilis*, *E. coli* and *S. cerevisiae*. MPGA = malt peptone glucose agar, YMG = yeast extract malt, PDA = potato dextrose agar, MEA = malt extract agar, YM = yeast malt agar, HBS = highly's basal salt agar. Error bars indicates the standard deviation of three technical replicates measurement for both fungal colony diameter (column in blue) and inhibition zone diameter (column in red).

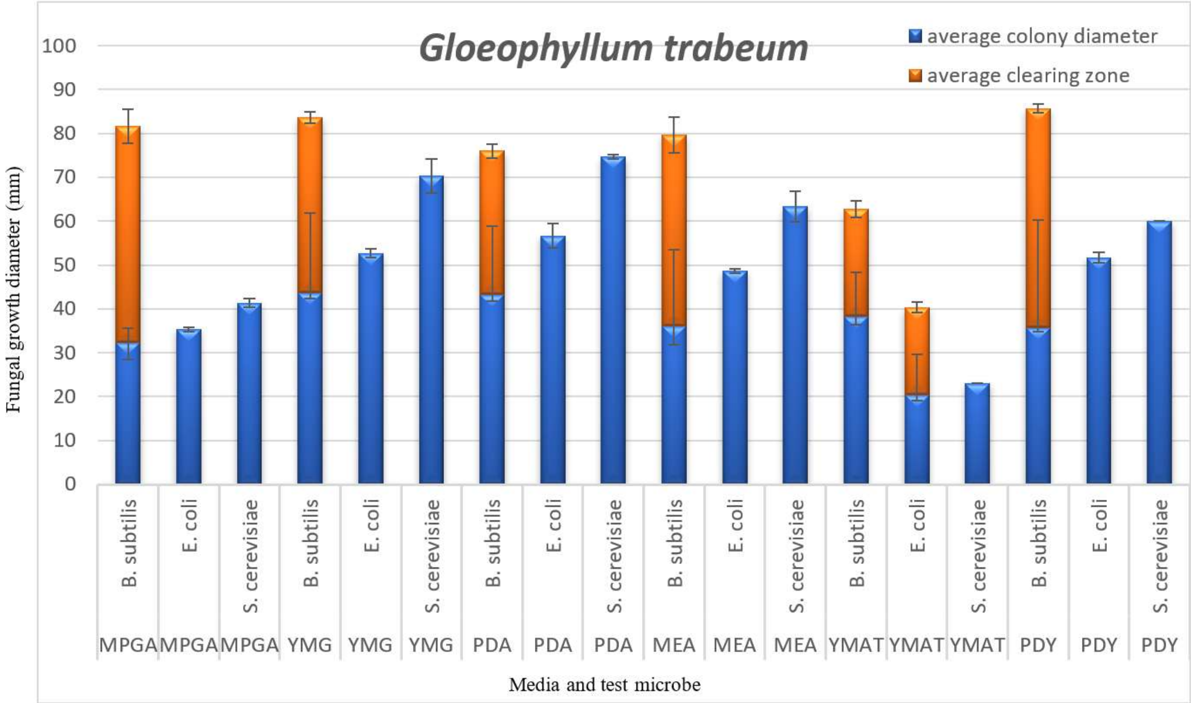

Figure S6: Bioassay test to evaluate the antimicrobial activity of *G. trabeum* growing on different media against *B. subtilis*, *E. coli* and *S. cerevisiae*. MPGA = malt peptone glucose agar, YMG = yeast extract malt, PDA = potato dextrose agar, MEA = malt extract agar, YMAT = yeast malt dextrose peptone agar, PDY = potato dextrose yeast extract. Error bars indicates the standard deviation of three technical replicates measurement for both fungal colony diameter (column in blue) and inhibition zone diameter (column in red).

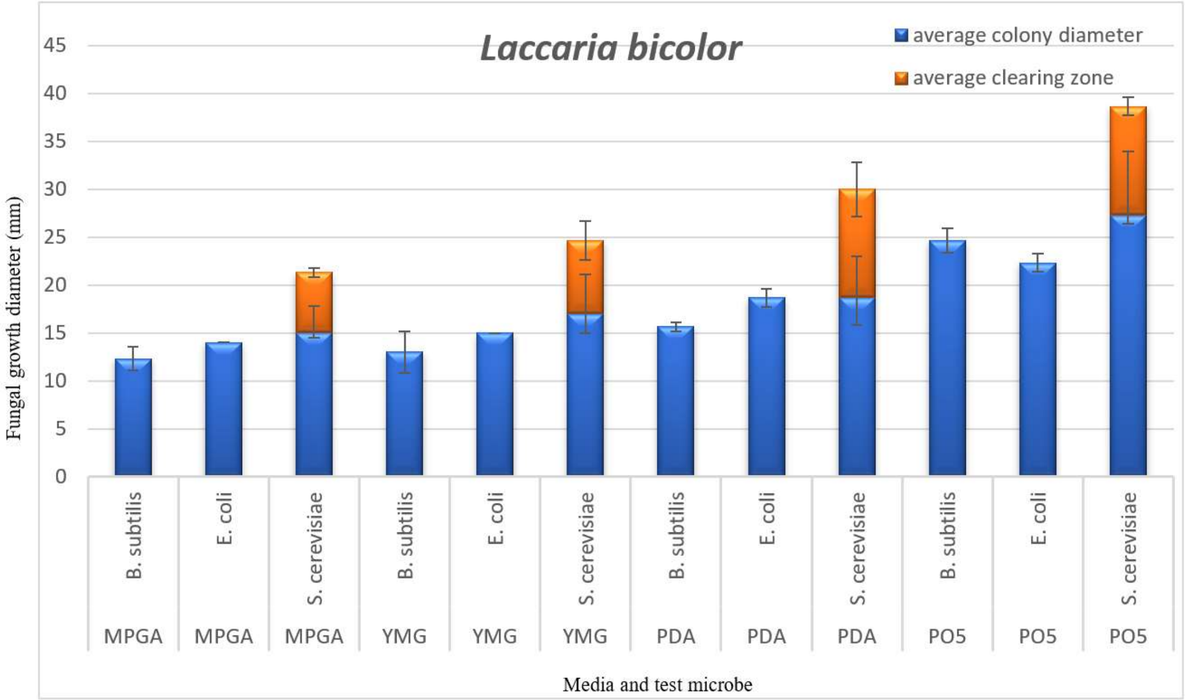

Figure S7: Bioassay test to evaluate the antimicrobial activity of *L. bicolor* growing on different media against *B. subtilis*, *E. coli* and *S. cerevisiae*. MPGA = malt peptone glucose agar, YMG = yeast extract malt, PDA = potato dextrose agar, PO5 = Pachlewski modified agar. Error bars indicates the standard deviation of three technical replicates measurement for both fungal colony diameter (column in blue) and inhibition zone diameter (column in red).

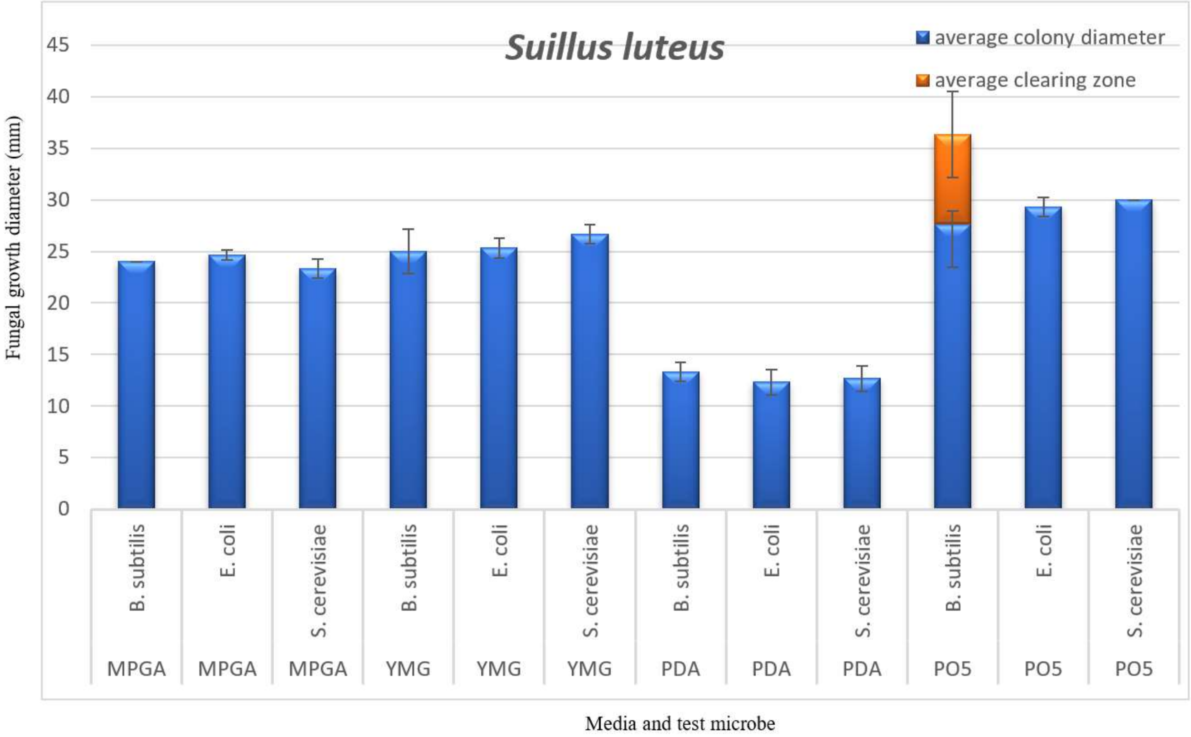

Figure S8: Bioassay test to evaluate the antimicrobial activity of *S. luteus* growing on different media against *B. subtilis*, *E. coli* and *S. cerevisiae*. MPGA = malt peptone glucose agar, YMG = yeast extract malt, PDA = potato dextrose agar, PO5 = Pachlewski modified agar. Error bars indicates the standard deviation of three technical replicates measurement for both fungal colony diameter (column in blue) and inhibition zone diameter (column in red).

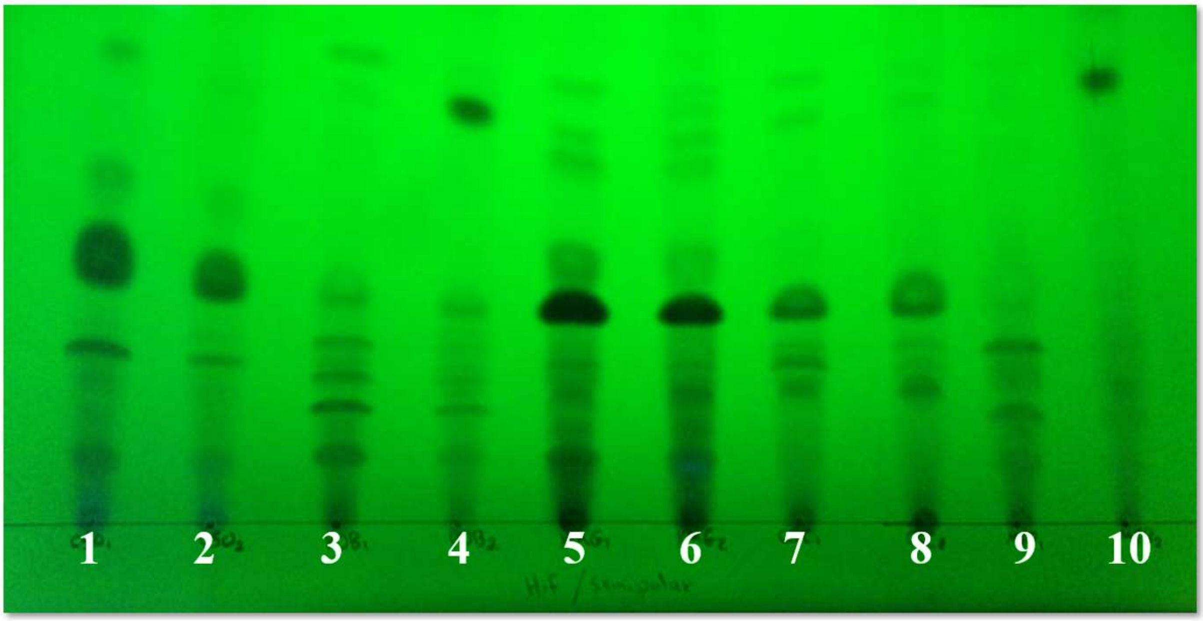

Figure S9: TLC plates developed in semi-polar system and visualized under UV 320 nm showing separated compounds of *H. fasciculare*. 25 µl of 10 mg/ml^{-1} of crude extract of *H. fasciculare* from five different media were spotted.
1 = CSO supernatant extract, 2 = CSO mycelial extract, 3 = PDB supernatant extract, 4 = PDB mycelial extract, 5 = YMG supernatant extract, 6 = YMG mycelial extract, 7 = CGC supernatant extract, 8 = CGC mycelial extract, 9 = MEB supernatant extract, 10 = MEB mycelial extract.

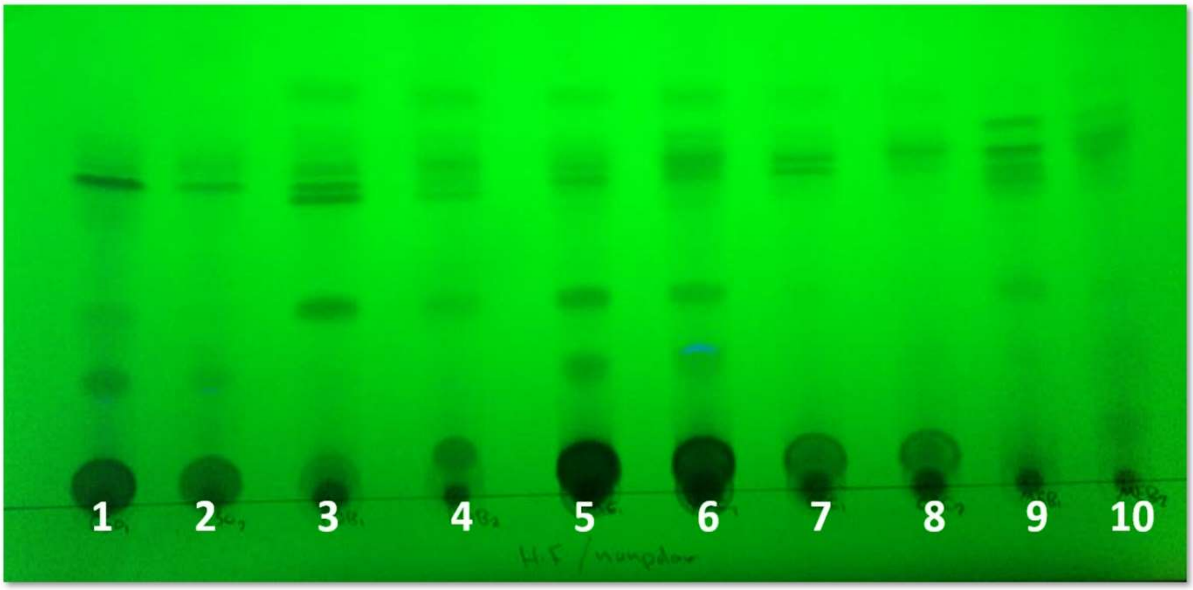

Figure S10: TLC plates developed in non-polar system and visualized under UV 320 nm showing separated compounds of *H. fasciculare*. 25 µl of 10 mg/ml^{-1} of crude extract of *H. fasciculare* from five different media were spotted.
1 = CSO supernatant extract, 2 = CSO mycelial extract, 3 = PDB supernatant extract, 4 = PDB mycelial extract, 5 = YMG supernatant extract, 6 = YMG mycelial extract, 7 = CGC supernatant extract, 8 = CGC mycelial extract, 9 = MEB supernatant extract, 10 = MEB mycelial extract.

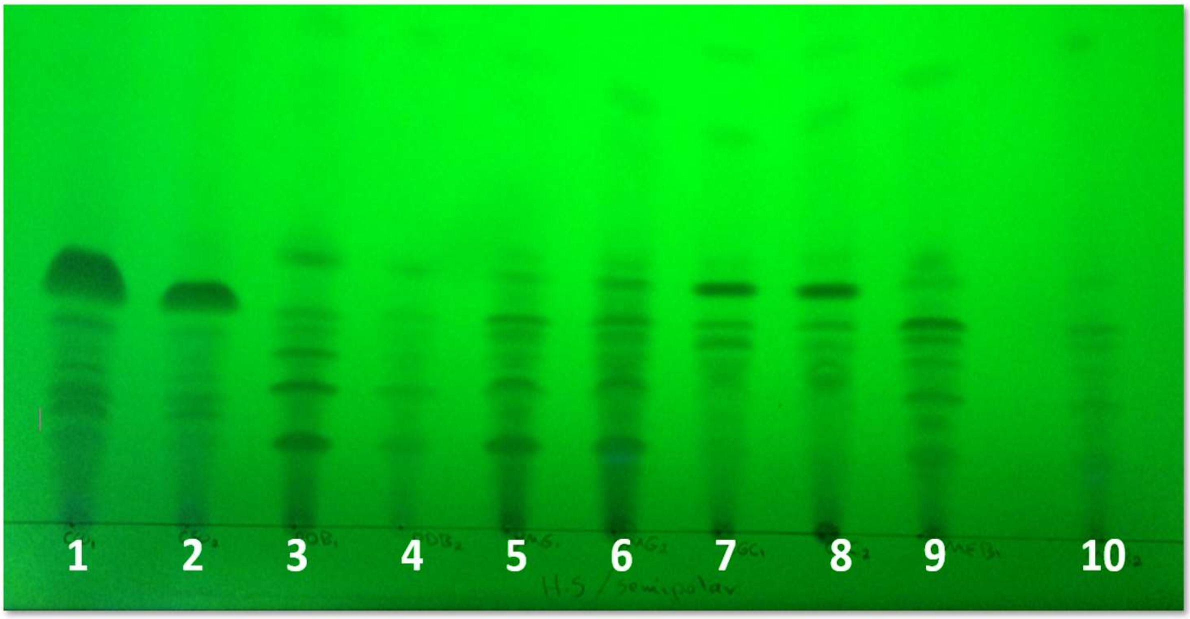

Figure S11: TLC plates developed in semi-polar system and visualized under UV 320 nm showing separated compounds of *H. sublateritium*. 25 µl of 10 mg/ml^{-1} of crude extract of *H. fasciculare* from five different media were spotted.
1 = CSO supernatant extract, 2 = CSO mycelial extract, 3 = PDB supernatant extract, 4 = PDB mycelial extract, 5 = YMG supernatant extract, 6 = YMG mycelial extract, 7 = CGC supernatant extract, 8 = CGC mycelial extract, 9 = MEB supernatant extract, 10 = MEB mycelial extract.

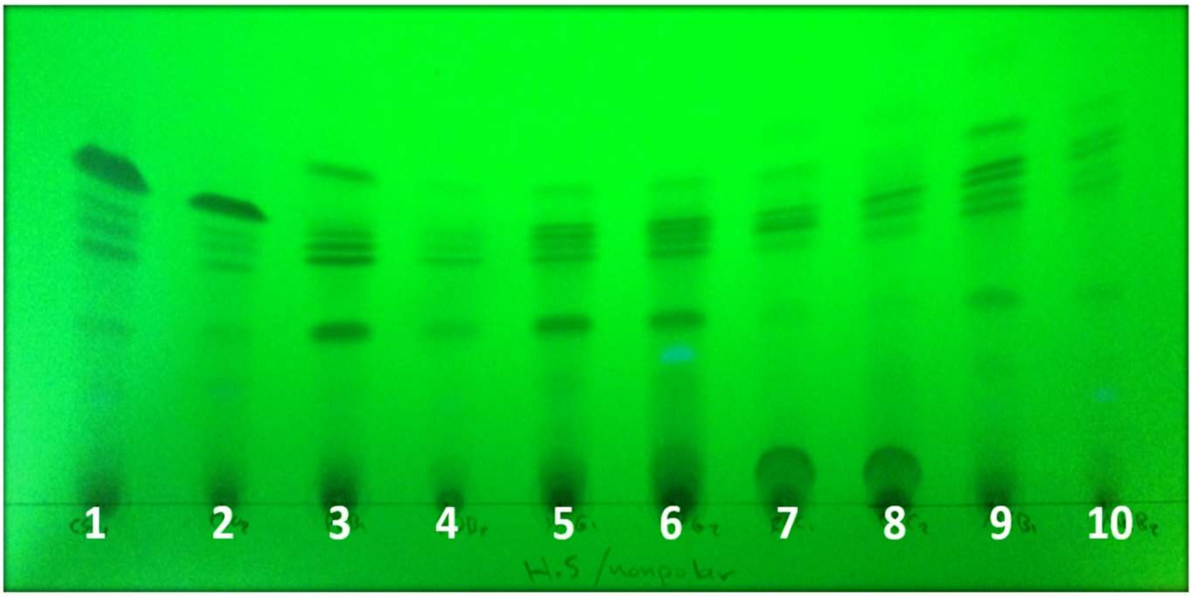

Figure S12: TLC plates developed in non-polar system and visualized under UV 320 nm showing separated compounds of *H. sublateritium*. 25 µl of 10 mg/ml^{-1} of crude extract of *H. fasciculare* from five different media were spotted.
1 = CSO supernatant extract, 2 = CSO mycelial extract, 3 = PDB supernatant extract, 4 = PDB mycelial extract, 5 = YMG supernatant extract, 6 = YMG mycelial extract, 7 = CGC supernatant extract, 8 = CGC mycelial extract, 9 = MEB supernatant extract, 10 = MEB mycelial extract.

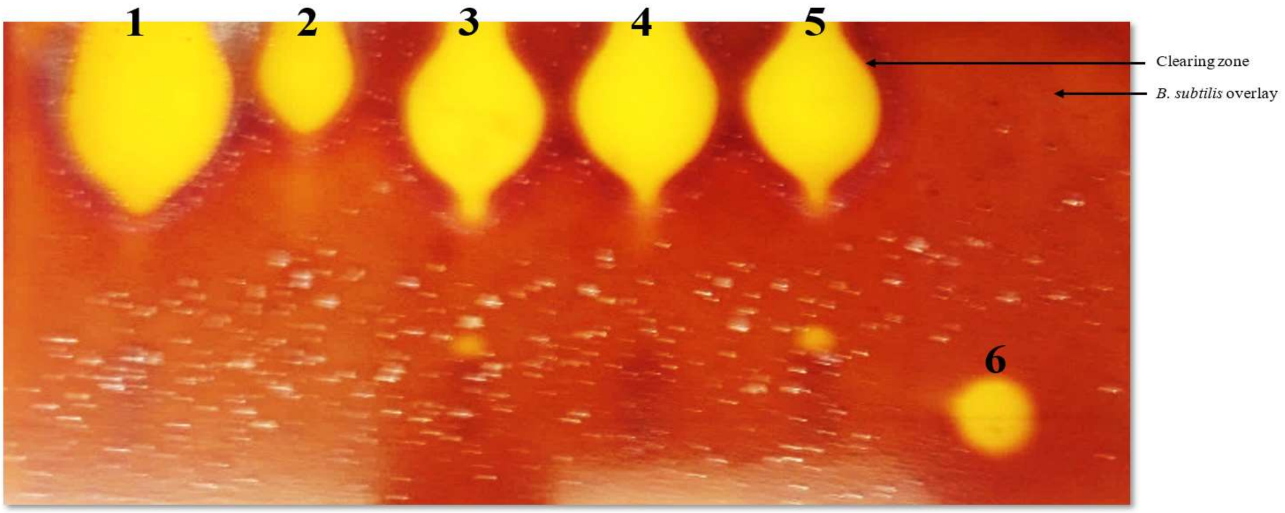

Figure S13: Direct bioautography of *H. fasciculare* crude extracts showing the antimicrobial activity of different culture crude extracts. 3 µl of 100 mg/ml^{-1} of mixed crude extract (supernatant+mycelia) of *H. fasciculare* of 1 = YMG, 2 = CSO, 3 = PDB, 4 = CGC, 5 = MEB media and 6 = 2 µl of 50 mg/ml of Kanamycin (positive control) loaded on TLC plates (developed in polar solvents) against *B. subtilis*.

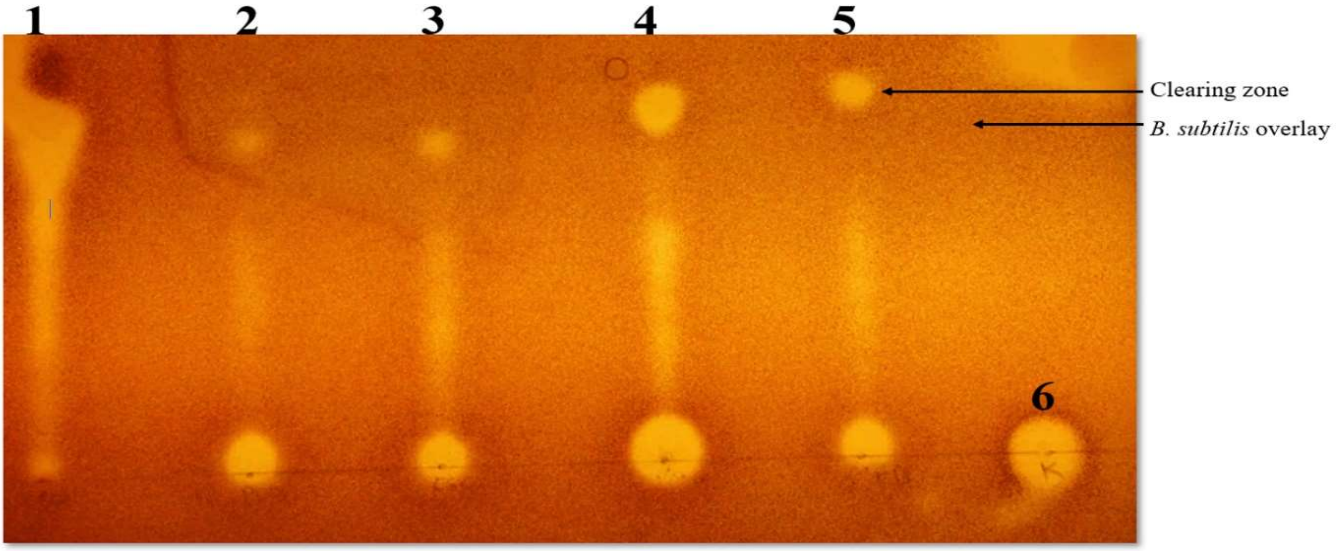

Figure S14: Direct bioautography of *H. fasciculare* crude extracts showing the antimicrobial activity of different culture crude extracts. 3 µl of 100 mg/ml^{-1} of mixed crude extract (supernatant+mycelia) of *H. fasciculare* of 1 = CSO, 2 = PDB, 3 = CGC, 4 =YMG, 5 = MEB media and 6 = 2 µl of 50 mg/ml of Kanamycin (positive control) loaded on TLC plates (developed in semi-polar solvents) against *B.*

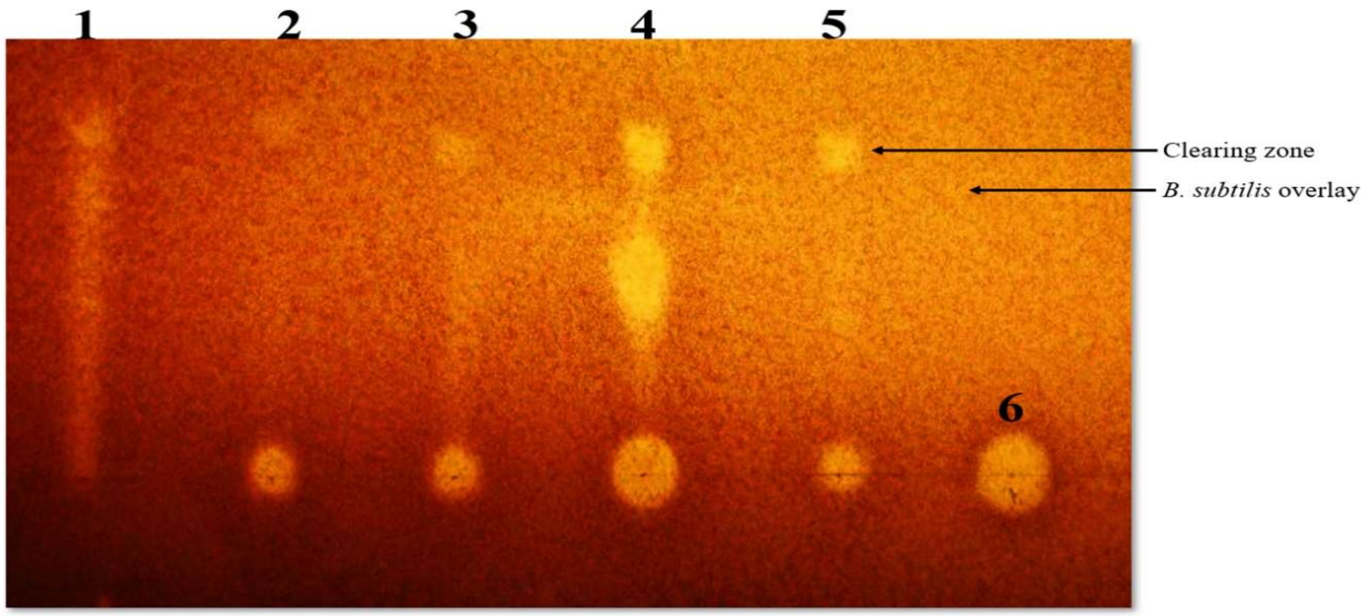

Figure S15: Direct bioautography of *H. fasciculare* crude extracts showing the antimicrobial activity of different culture crude extracts. 3 µl of 100 mg/ml^{-1} of mixed crude extract (supernatant+mycelia) of *H. fasciculare* of 1 = CSO, 2 = PDB, 3 = CGC, 4 =YMG, 5 = MEB media and 6 = 2 µl of 50 mg/ml of Kanamycin (positive control) loaded on TLC plates (developed in non-polar solvents) against *B. subtilis*.

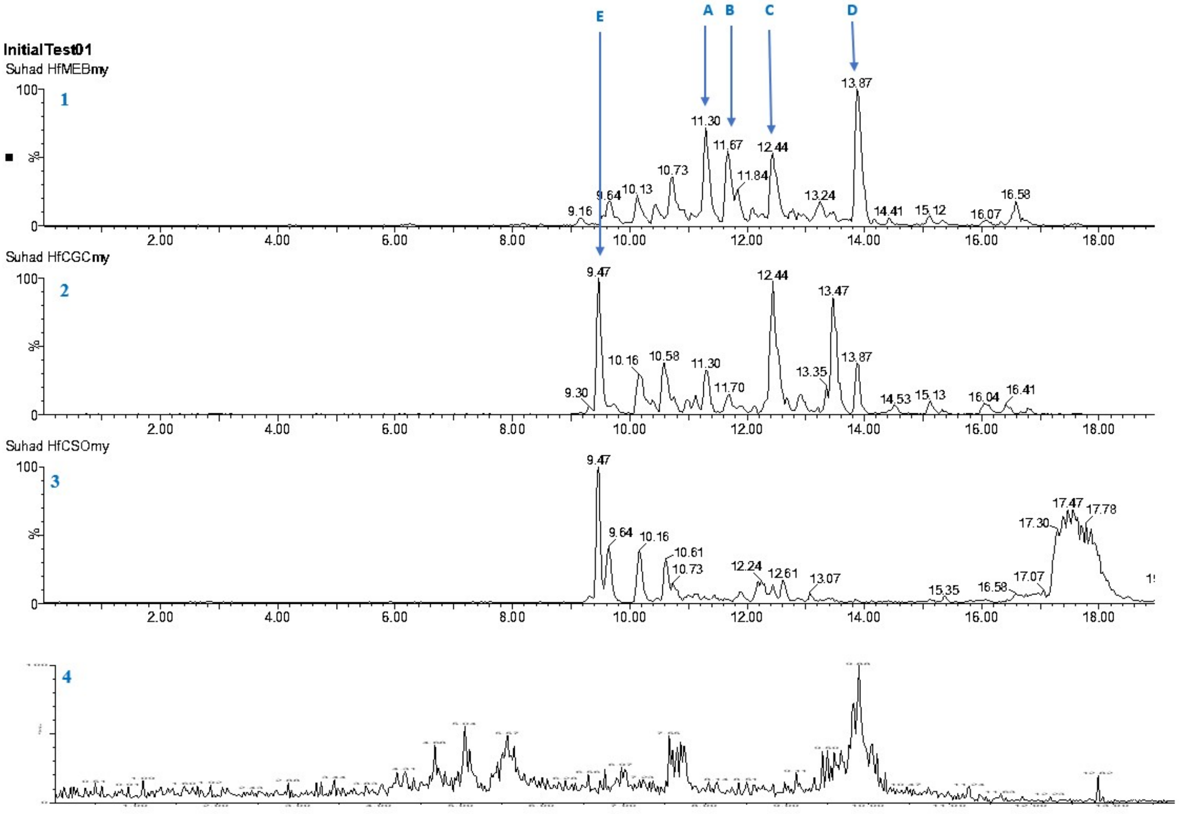

Figure S16: LC-MS chromatograms electrospray mass negative mode (ES-) of 5 mg/ml of:

1- *H. fasciculare* MEB crude extract. 2- *H. fasciculare* CGC crude extract. 3- *H. fasciculare* CSOA-1 crude extract. 4- *H. fasciculare* YMG crude extract

A = Facicularone G. B = naematolin. C = *Unknow*. D = Unknown. E = *Unknow*.

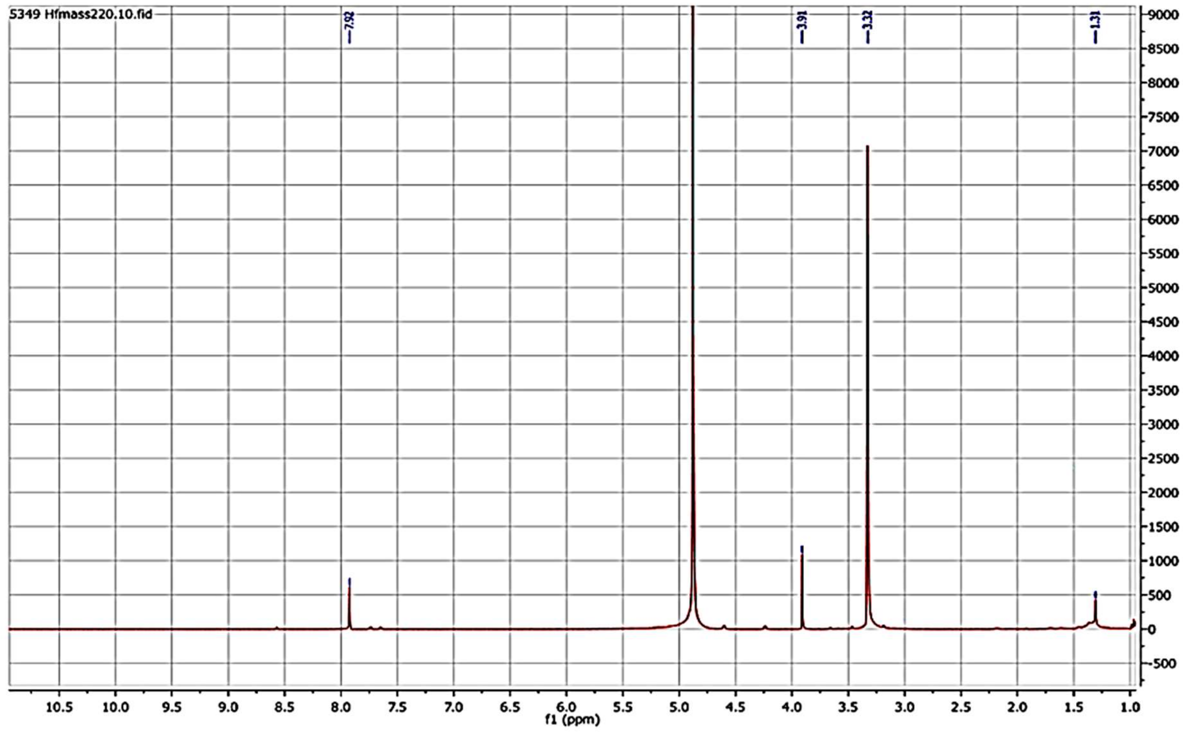

Figure S17: ^{1}H NMR (125MHZ) spectrum of 3,5-dichloro-4-methoxybenzoic acid (3, 5-D).

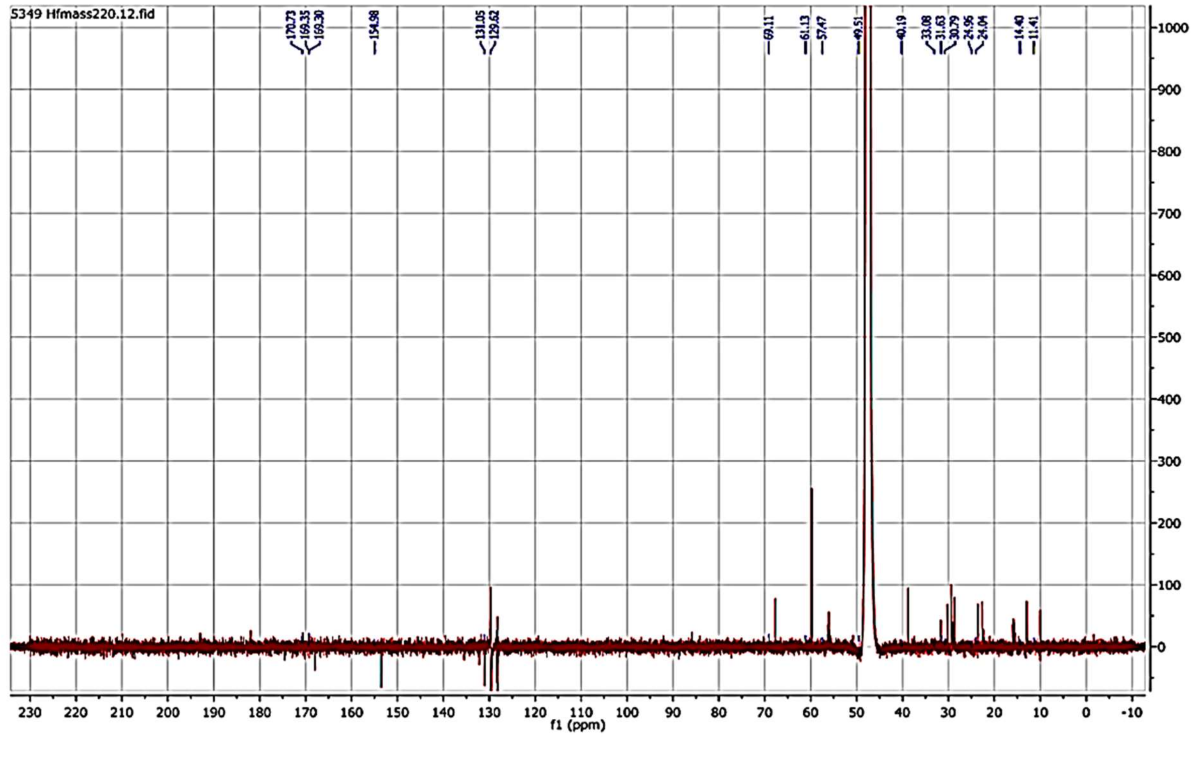

Figure S18: ^{13}C NMR (500MHZ) spectrum of 3,5-dichloro-4-methoxybenzoic acid (3, 5-

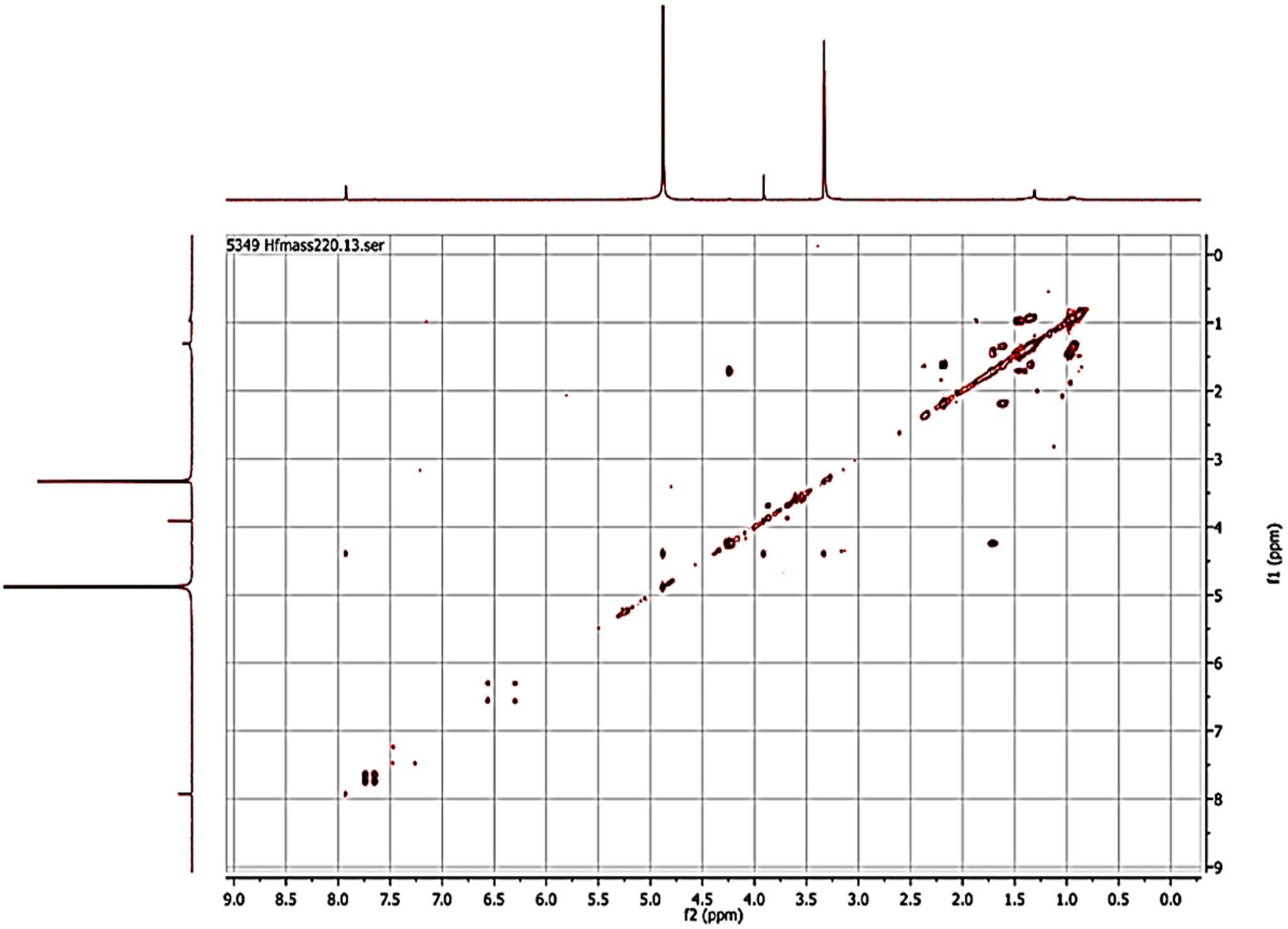

Figure S19: COSY (500MHZ) spectrum of 3,5-dichloro-4-methoxybenzoic acid (3, 5-D).

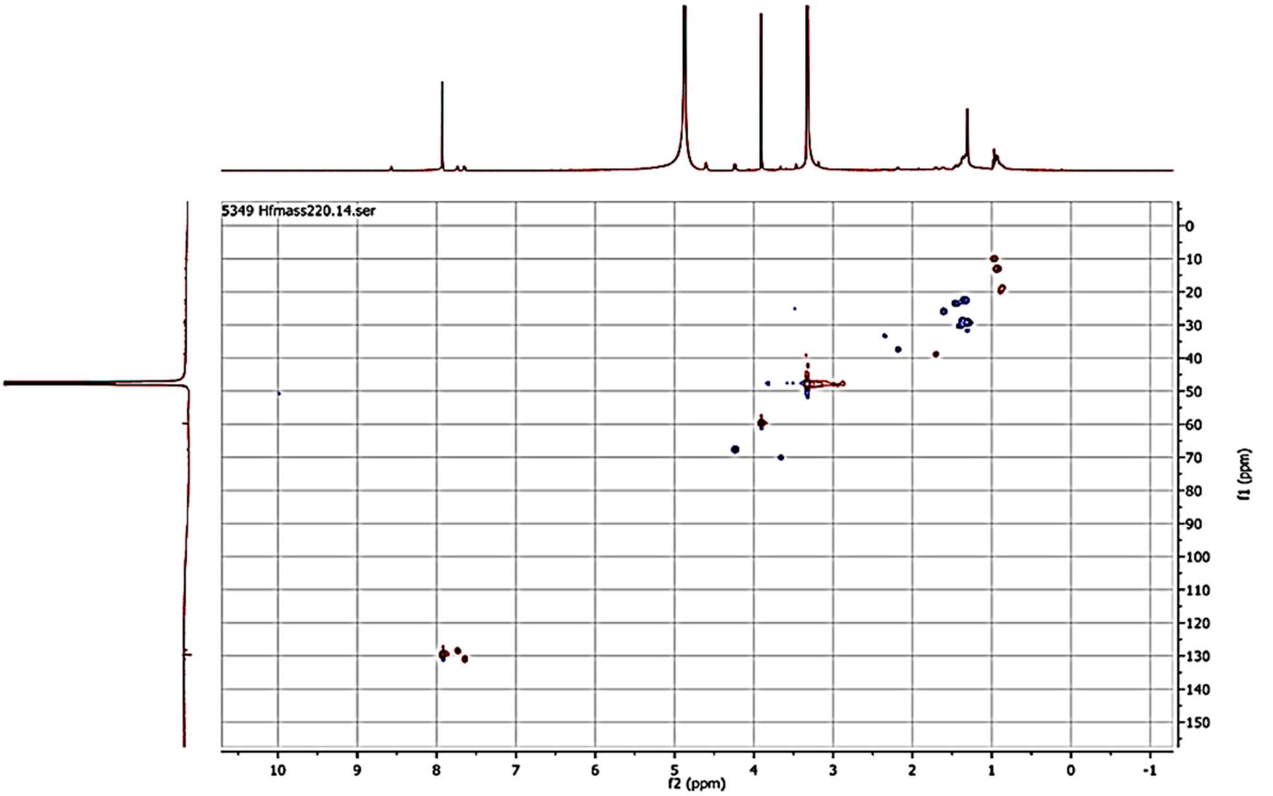

Figure S20: HSQC (500MHZ) spectrum of 3,5-dichloro-4-methoxybenzoic acid (3, 5-D).

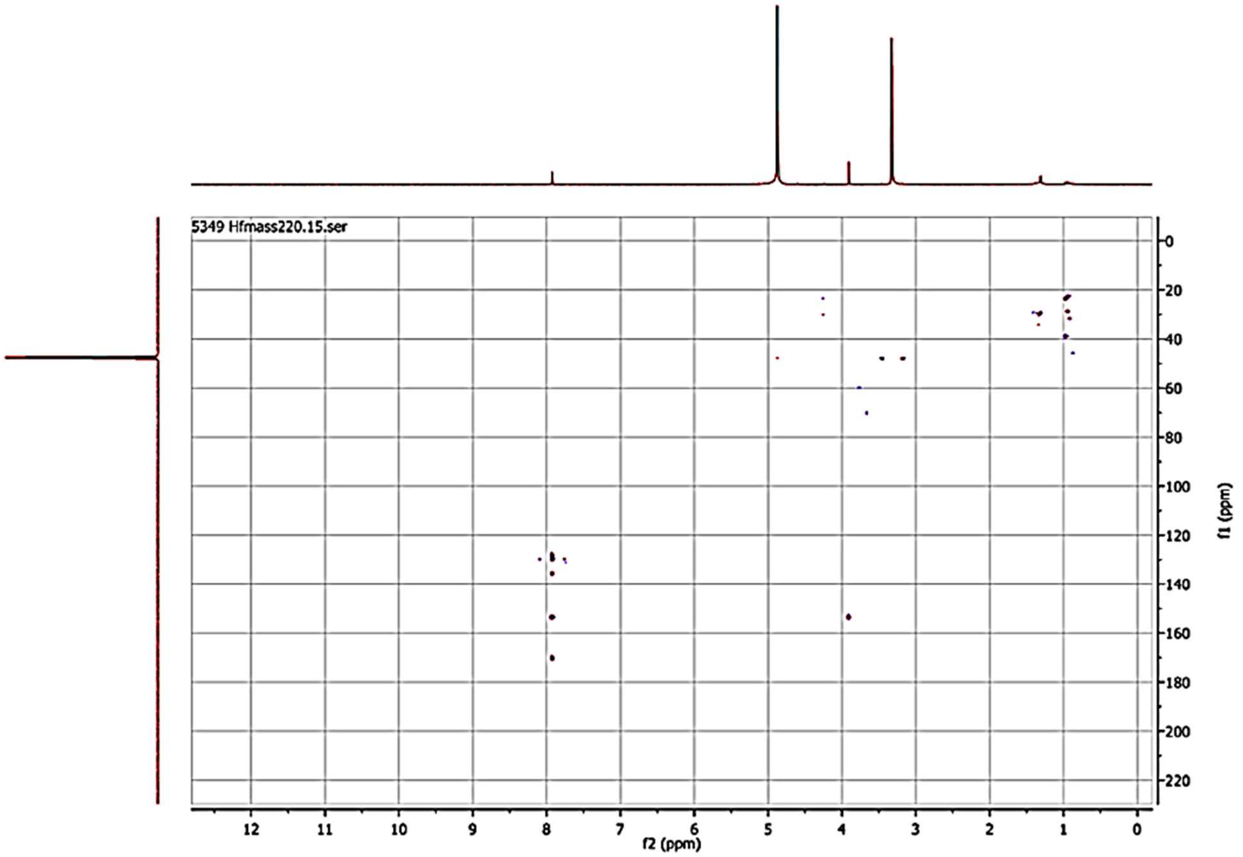

Figure S21: HMBC (500MHZ) spectrum of 3,5-dichloro-4-methoxybenzoic acid (3, 5-D).

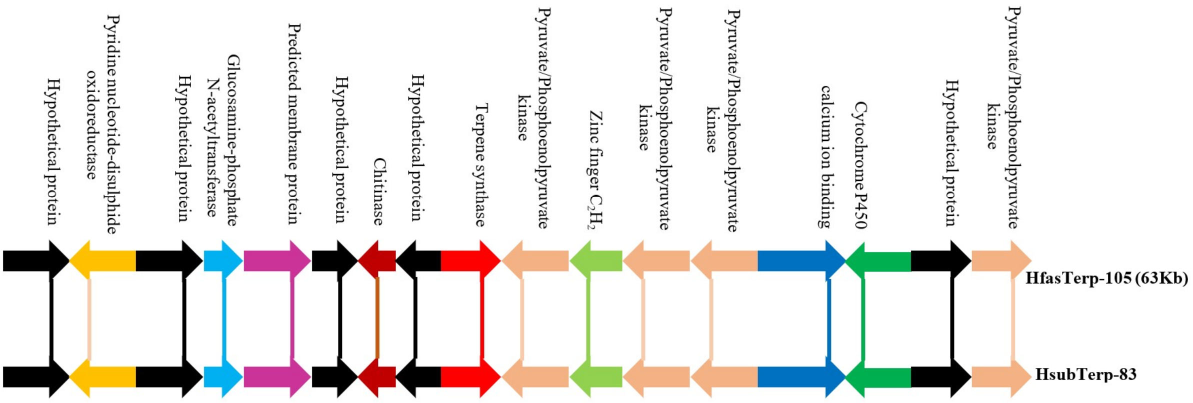

Figure S22: Predicted gene cluster of Hfas-terp105 and the homologous cluster (Hsub-terp83) in *H. sublateritium*.

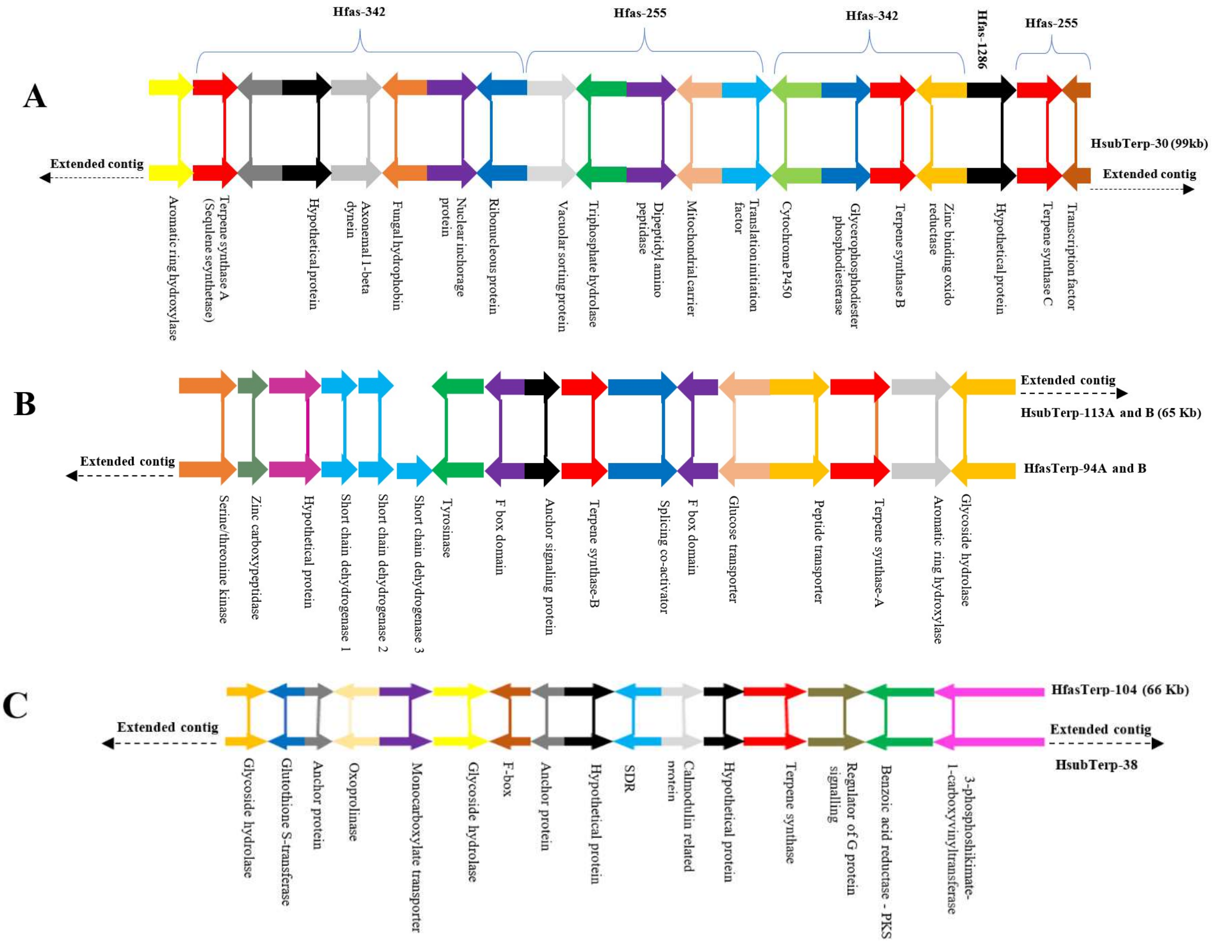

Figure S23: Predicted gene cluster of *H. sublateritium* terpene synthases and *H. fasciculare*.

A. *H. sublateritium* scaffold 30 terpene synthase gene clusters and the homologous clusters of *H. fasciculare*.
B. *H. sublateritium* scaffold 113 terpene synthase gene clusters and the homologous clusters of *H. fasciculare*.
C. *H. sublateritium* scaffold 38 terpene synthase gene clusters and the homologous clusters of *H. fasciculare*.

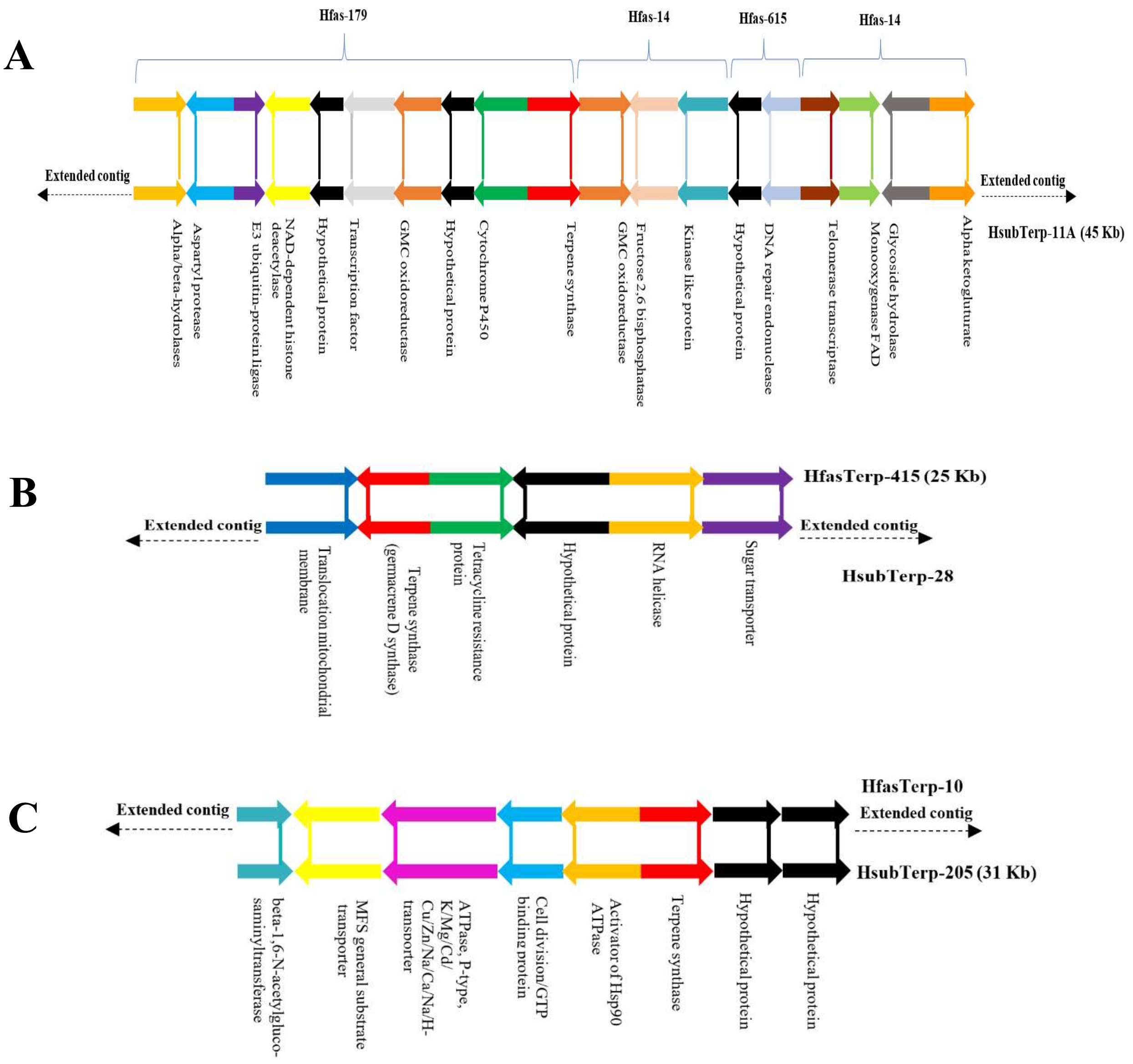

Figure S24: Predicted gene cluster of *H. sublateritium* terpene synthases and *H. fasciculare*.

A. *H.* sublateritium scaffold 11A terpene synthase gene clusters and the homologous clusters of *H. fasciculare*.
B. *H. sublateritium* scaffold 205 terpene synthase gene clusters and the homologous clusters of *H. fasciculare*.
C. *H. sublateritium* scaffold 28 terpene synthase gene clusters and the homologous clusters of *H. fasciculare*.

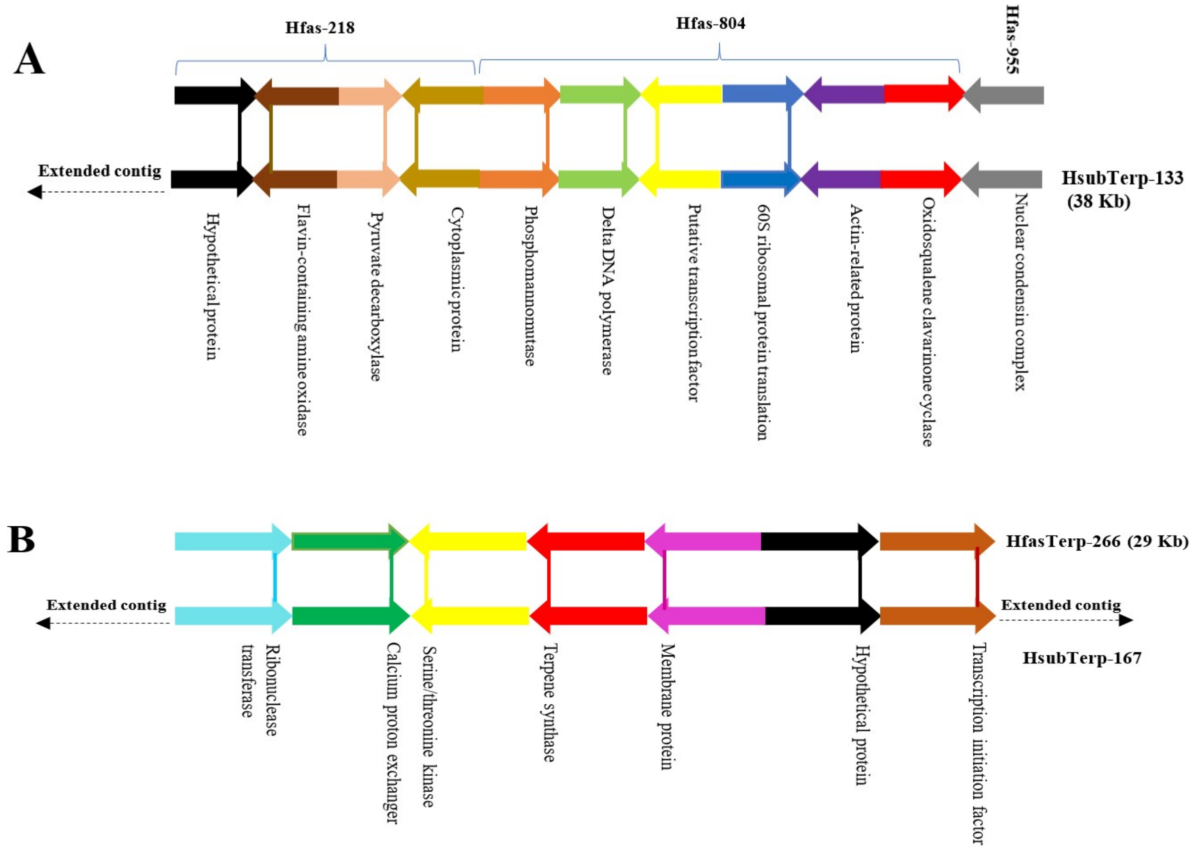

Figure S25: Predicted gene cluster of *H. sublateritium* terpene synthases and *H. fasciculare*.

A. *H. sublateritium* scaffold 133 terpene synthase gene clusters and the homologous clusters of *H. fasciculare*.
B. *H. sublateritium* scaffold 167 terpene synthase gene clusters and the homologous clusters of *H. fasciculare*.

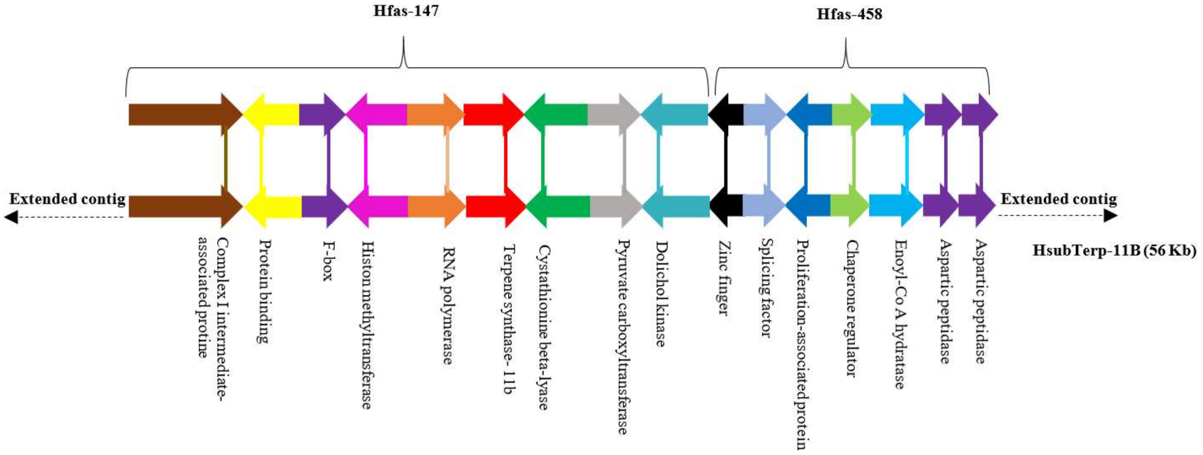

Figure S26: Predicted gene cluster of *H. sublateritium* terpene synthase 11B and the homologous cluster in *H. fasciculare*.

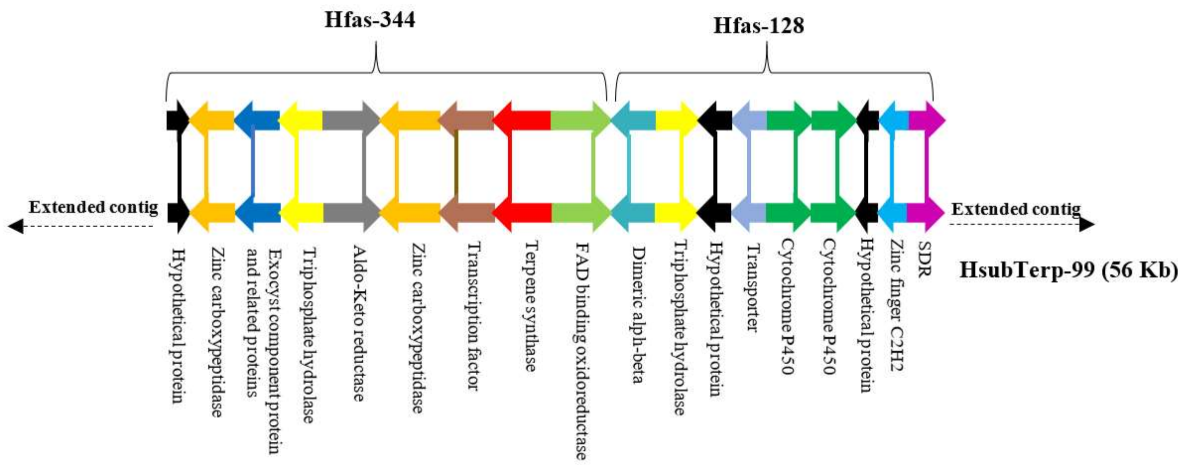

Figure S27: Predicted gene cluster of *H. sublateritium* terpene synthase 99 and the homologous cluster in *H. fasciculare*.

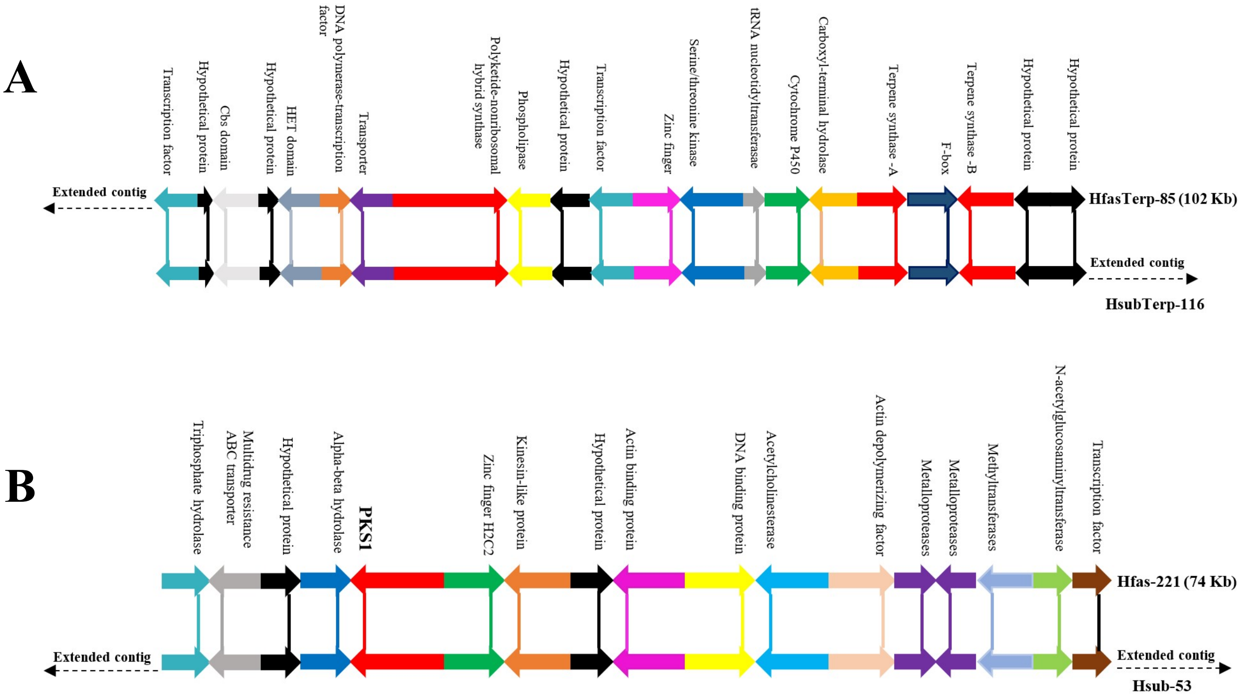

Figure S28: Predicted gene cluster of *H. fasciculare* and *H. sublateritium* PKS.

A. *H. fasciculare* PKS 102 biosynthetic gene cluster and the homologous cluster of *H. sublateritium.*

B. *H. fasciculare* PKS 221 biosynthetic gene cluster and the homologous cluster of *H. sublateritium.*

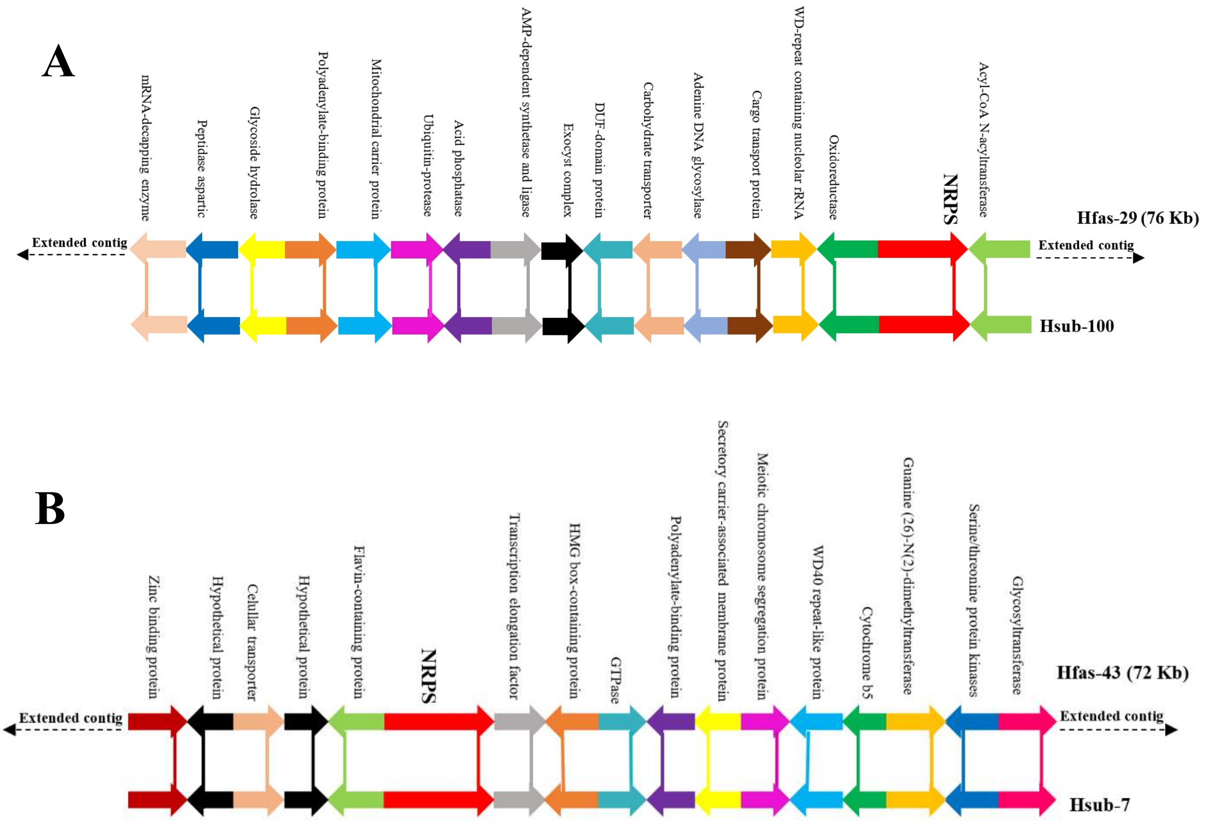

Figure S29: Predicted gene cluster of *H. fasciculare* and *H. sublateritium* NRPS.

A. *H. fasciculare* NRPS 29 biosynthetic gene cluster and the homologous cluster of *H. sublateritium*.
B. *H. fasciculare* NRPS 43 biosynthetic gene cluster and the homologous cluster of *H. sublateritium*.

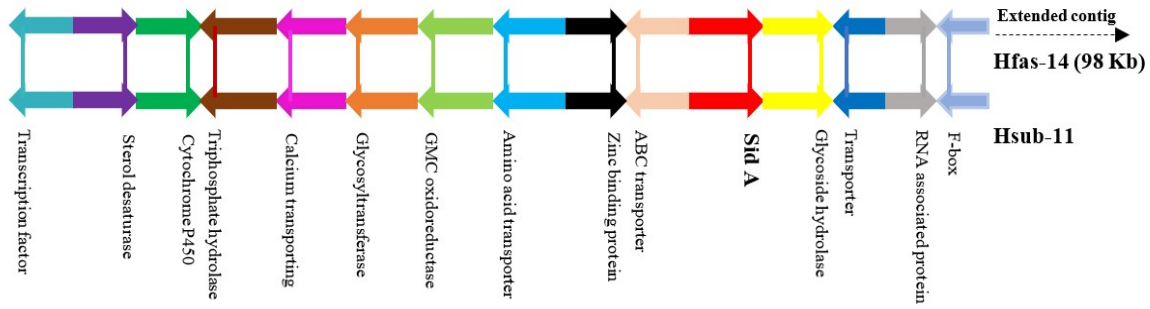

Figure S30: Predicted gene cluster of *H. sublateritium* SidA and the homologous cluster in *H. fasciculare*.

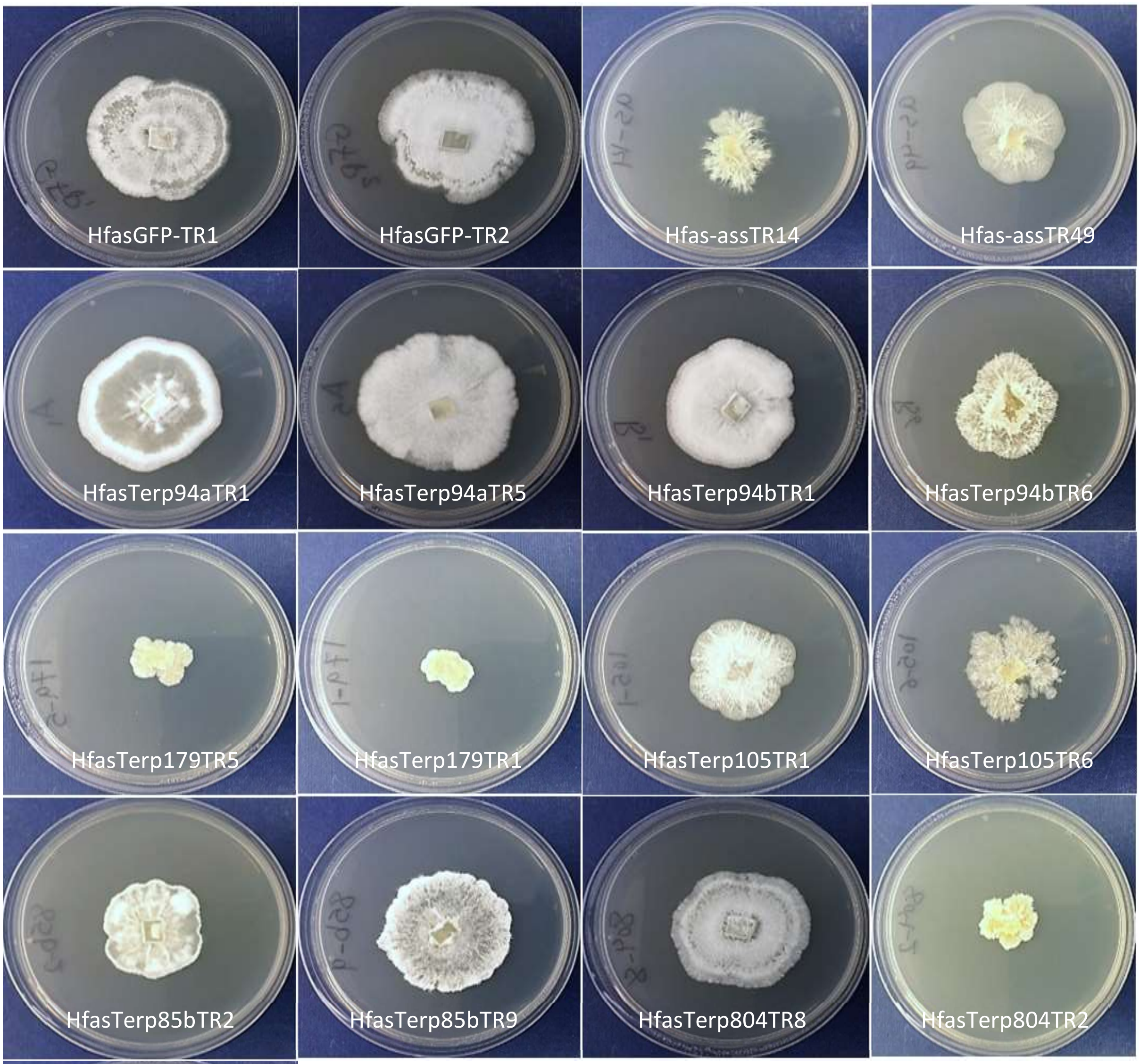

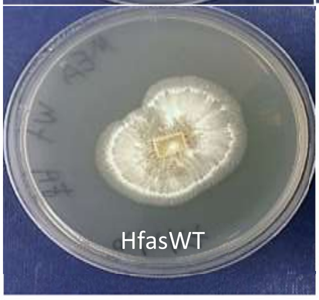

Figure S31: Represents randomly selected mutants of *H. fasciculare* terpene synthase gene silencing experiments along with two GFP transformants and the wild type. All selected colonies were cultured on PDA media for two weeks at 25°C. Mutants demonstrated variant levels of gene expression downregulation through the development of unusual patterns of growth; white fluffy mycelia, creamy sparse fine mycelia, and yellowish wrinkled mycelia. Transformants were similar phenotypically to the WT and showed white fluffy mycelia included GFP transformants TR1 & TR2, terpene synthase 94A transformants TR1 & TR5, terpene synthase 94B transformant TR1, terpene synthase 85B transformants TR2 & TR9 & terpene synthase 804 transformant TR8. Transformants showed creamy sparse fine mycelia included argininosuccinate synthetase transformant TR14, terpene synthase 94B transformant TR6, terpene synthase 105 transformants TR1 & TR6. Transformants showed yellowish wrinkled mycelia included argininosuccinate synthetase transformant TR49, terpene synthase 179 transformants TR1 & TR5, terpene synthase 804 transformant TR2.

SAMEB

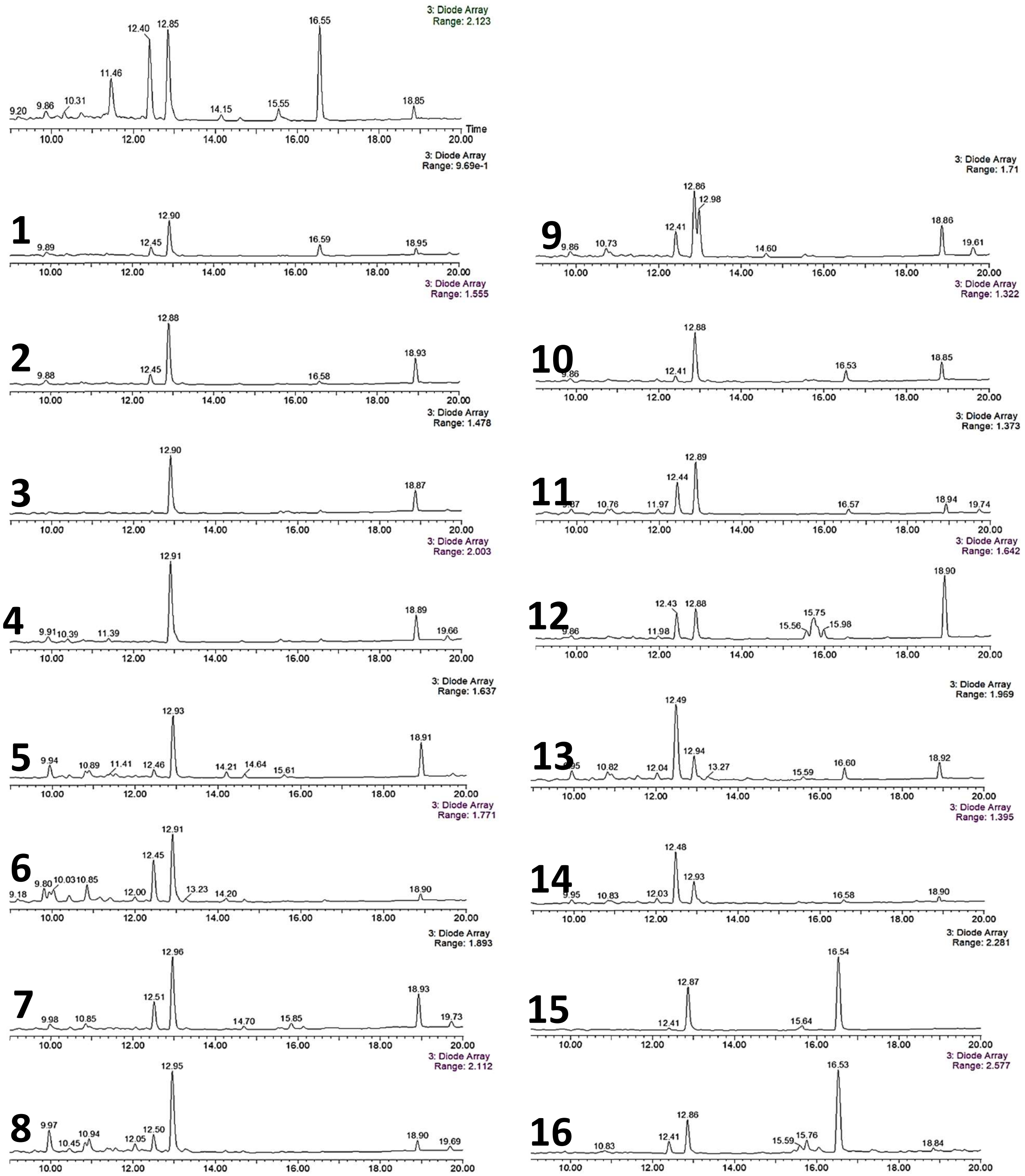

Figure S32: Diode array chromatograms for *H. fasciculare* WT and putative silenced transformants crude extracts. The production of main secondary metabolites was compared in the WT with two transformants of each silenced line. The genes investigated in this experiment were, argininosuccinate synthase and seven terpene synthases from different biosynthetic clusters. SAMEB = Hfas WT. 1 = Hfas-assTR14, 2- Hfas-assTR49, 3- HfasTerp85bTR2, 4- HfasTerp85bTR9, 5- HfasTerp94aTR1, 6- HfasTerp94aTR5, 7- HfasTerp94bTR1, 8- HfasTerp94bTR6, 9- HfasTerp105TR1, 10- HfasTerp105TR6, 11- HfasTerp179TR1, 12- HfasTerp179TR5, 13- HfasTerp342TR18, 14- HfasTerp342TR6, 15- HfasTerp804TR2, 16- HfasTerp804TR8.

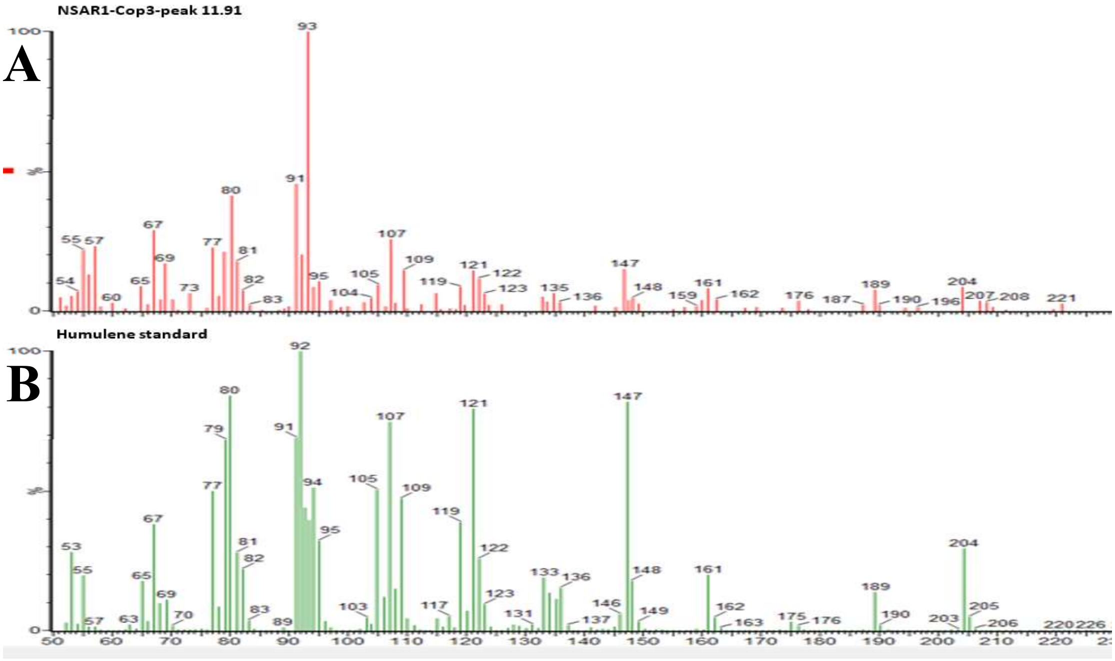

Figure S33: GC-MS spectrum comparison of A. Sesquiterpene synthase Cop3 and B. Humulene

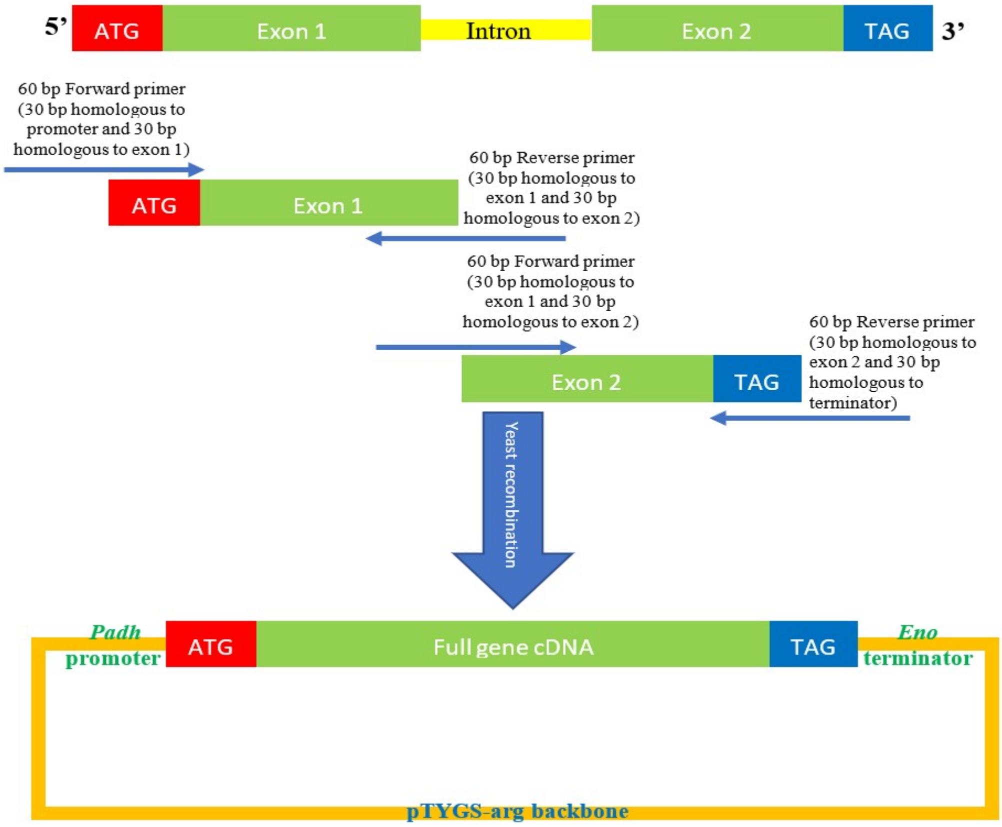

Figure S34: Schematic representation of the principle of constructing the pTYGS-arg-SDR plasmid. Each SDR gene consisted of two exons and one intron. Exons shown in green. Intron shown in yellow. Overlapping primers shown as blue arrows. pTYGS-arg backbone was digested with *Asc*I and the region between *Padh* and *Teno* was replaced by the created full cDNA of one of the SDR genes.

Table S3: List of primers used for *H. fasciculare* terpene synthase antisense plasmids construction

No.	Dir	Primer	Sequence (5'->3')	Fragment size(bp)	Description
**1**	**F**	pUC8MCS linker CaMVT terminator (pU-CaMVT-F)	TGTTGGGCCCGGCGCGCCGAATTCCCGGGA CTGGATTTTGGTTTTAGGAATTAGAAATTT	±1512	Amplify hygromycin cassette under *A. bisporus gpdII* promoter
**2**	**R**	*A. bisporus gpdII* promoter (A.gpdIIpr-R)	GAAGAAGAATTCAGAGGTCCGCAAGTAGAT		
**3**	**F**	*A. bisporus gpdII* promoter *Hypholoma gpd* promoter (A & *H.gpd* pr-F)	ATCTACTTGCGGACCTCTGAATTCTTCTTC GTACTTACTAGCCCATCTTTGGCTCTATCG	±1000	Amplify *Hs.gpd* promoter
**4**	**R**	*Hypholoma gpd* promoter (H.gpd pr-R)	ACAGCATCGAACTGGTTTGGTGGATAAATG		
**5**	**F**	*Hsgpd promoter Hf.aras gene (Hsgpd & Hf.aras.F)*	CATTTATCCACCAAACCAGTTCGATGCTGTA TGAGCCTGACCAAGCCGTTGACGGTGCGC	±630	Amplify Hf argininosuccinate synthase-antisense
**6**	**R**	*Hf.aras gene (Hf.aras.R)*	CTACAACCGCTTCCCCGGCCGCCAGGCGCTC		
**7**	**F**	*Hf.aras gene* and *A. nidulans trpC* terminator (H. & aras-F)	AGCGCCTGGCGGCCGGGGAAGCGGTTGTAG ATCCACTTAACGTTACTGAAATCATCAAAC	±900	Amplify *trpC* terminator for aras
**8**	**R**	*A. nidulans trpC* terminator nos (A.trpC-R)	CTCCTCTTAAAGCTTGGCTGCAGGTCGACG GCGGCCGCCAGTGTGATGGATATCTGCAGA		
**9**	**F**	*Hsgpd promoter Hf.94a gene (Hsgpd & Hf.94a.F)*	CATTTATCCACCAAACCAGTTCGATGCTGTA TGGCTATCAAATCAGTCGCACATTCCACC	±500	Amplify Hf Terp94a-antisense
**10**	**R**	*Hf.aras gene (Hf.94a.R)*	CATCTCCGGGTGGCATGCGACTTGATGAAT		
**11**	**F**	*Hf.94a gene* and *A. nidulans trpC* terminator (H. & 94a-F)	ATTCATCAAGTCGCATGCCACCCGGAGATGA TCCACTTAACGTTACTGAAATCATCAAAC	±900	Amplify *trpC* terminator for 94a
**12**	**F**	*Hsgpd promoter Hf.94b gene (Hsgpd & Hf.94b.F)*	CATTTATCCACCAAACCAGTTCGATGCTGTA TGTTAAATCAGTTGCACTTTCTGTCAAGG ATT	±500	Amplify Hf Terp94b-antisense
**13**	**R**	*Hf.aras gene (Hf.94b.R)*	GCGCATATCATGTGATTTGATGAATTTCTACT T		
**14**	**F**	*Hf.94a gene* and *A. nidulans trpC*	TAGAAATTCATCAAATCACATGATATGCGCA TCCACTTAACGTTACTGAAATCATCAAAC	±900	Amplify *trpC* terminator for 94b

		terminator (H. & 94b-F)			
**15**	**F**	*Hsgpd promoter Hf.105 gene (Hsgpd & Hf.105.F)*	CCAAACCAGTTCGATGCTGTATGGGAATCTT TTTCCACTCCTCTAGAAATTGCGCTTCTG	±500	Amplify Hf Terp105-antisense
**16**	**R**	*Hf.aras gene (Hf.105.R)*	GGTTGAATGCAAGAAGATTTGCAACGCTCT		
**17**	**F**	*Hf.94a gene* and *A. nidulans trpC* terminator (H.&105-F)	AGAGCGTTGCAAATCTTCTTGCATTCAACCA TCCACTTAACGTTACTGAAATCATCAAAC	±900	Amplify *trpC* terminator for 105
**18**	**F**	*Hsgpd promoter Hf.85b gene (Hsgpd & Hf.85b.F)*	TTATCCACCAAACCAGTTCGATGCTGTATGG CCTGCCTTTGTGCTGCCGCCAAAGACCCA	±600	Amplify Hf Terp85b-antisense
**19**	**R**	*Hf.85b gene (Hf.85b.R)*	CCATCACGCCGACCGTAGAGCGGCTCGCGG		
**20**	**F**	*Hf.85b gene* and *A. nidulans trpC* terminator (H.&85b-F)	CCGCGAGCCGCTCTACGGTCGGCGTGATGGA TCCACTTAACGTTACTGAAATCATCAAAC AGCT	±900	Amplify *trpC* terminator for 85b
**21**	**F**	*Hsgpd promoter Hf.804 gene (Hsgpd & Hf.804.F)*	TTATCCACCAAACCAGTTCGATGCTGTATGT CGCGAAGCCGCCATTGGGATTCTGCATGG TGAG	±1000	Amplify Hf Terp804-antisense
**22**	**R**	*Hf.804 gene (Hf.804.R)*	GGTCCCTTTCCATCCGAGCAAGTGGTGGAT		
**23**	**F**	*Hf.804gene* and *A. nidulans trpC* terminator (H.&804-F)	ATCCACCACTTGCTCGGATGGAAAGGGACCA TCCACTTAACGTTACTGAAATCATCAAAC	±900	Amplify *trpC* terminator for 804
**24**	**F**	*Hsgpd promoter Hf.342 gene (Hsgpd & Hf.342.F)*	TTATCCACCAAACCAGTTCGATGCTGTATGC GCGGAAGATGAGGCCGACTTCCCGGGGAT TCGT	±1000	Amplify Hf Terp342-antisense
**25**	**R**	*Hf.342 gene (Hf.342.R)*	CTATTCTATCTAGTCCTTCGCGGACTAGAC		
**26**	**F**	*Hf.342gene* and *A. nidulans trpC* terminator (H. & 342-F)	GTCTAGTCCGCGAAGGACTAGATAGAATAGA TCCACTTAACGTTACTGAAATCATCAAAC AGCT	±900	Amplify *trpC* terminator for 342
**27**	**F**	*Hsgpd promoter Hf.179 gene (Hsgpd & Hf.179.F)*	TTATCCACCAAACCAGTTCGATGCTGTATGG GGATATATAACTCTCGGGGTCTACTATGT	±500	Amplify Hf Terp179-antisense
**28**	**R**	*Hf.179 gene (Hf.179.R)*	GAATTATCAGTTCGGAGTCCAAGCAATGGT		
**29**	**F**	*Hf.179gene* and *A. nidulans trpC* terminator (H.&179-F)	ACCATTGCTTGGACTCCGAACTGATAATTCA TCCACTTAACGTTACTGAAATCATCAAAC	±900	Amplify trpC terminator for 179

Materials and Methods

Bioassays

Plate based bioassay

Preparing of *Bacillus subtilis* spores' suspension

The method of Hartley et al. (2009) was used to prepare a *B. subtilis* spore suspension, in which glycerol stock of *Bacillus* was used to inoculate 50 ml culture of tryptic soy broth (TSB), and this was incubated for 24 hours at 30°C with shaking at 200 rpm. 1ml of this overnight culture was spread with sterile glass beads on the surface of tryptic soy agar slant (prepared by adding 75 ml of tryptic soy agar (TSAb) to Thompson bottle, and allowed to set horizontally after autoclaving. The cells were harvested after 6 days incubation at 30°C by using again sterile glass beads and 100 ml of sterile deionised water. *Bacillus* suspension was then heated at 70°C in a water bath for 30 minutes to activate the spores and kill the vegetative cells. These spores were subsequently stored at 4°C until required.

Preparation of overlays

B. subtilis overlay was prepared by adding 800 µl of *B. subtilis* spore suspension to 400 ml molten overlay medium (TSAg 70°C) to heat shock the spores. To visualize the overlain bacteria, 2 ml of 0.4% 2,3,5-triphenyl-2H-tetrazolium chloride (TTC) was added to the cooled agar (approximately 50°C), before pouring the overlay.

For *E. coli* overlay preparation, 5 ml of LB broth was inoculated with a colony from a fresh DH5α plate, before incubating overnight at 200 rpm at 37°C. This culture was then transferred to a sterile Erlenmeyer flask containing 100 ml of fresh LB medium and incubated at 200 rpm, 37°C until the optical density OD600 of the culture reached 1.0. 2 ml of the bacterial suspension and 0.4% of 2,3,5-triphenyl-2H-tetrazolium chloride (TTC) were then added to 1L of the molten (approximately 50°C) overlay medium LBA.

The *S. cerevisiae* culture was prepared by inoculating 5 ml of YPD medium with a yeast colony (Y10000), before being shaken at 200 rpm at 28°C for 24 hours. The overnight culture was then transferred to 100 ml of YPD broth and shaken as before. Once the optical density OD600 reached 1.0, 2 ml of this culture and 0.4% 2,3,5-triphenyl-2H-tetrazolium chloride (TTC) were added to 1 L of the molten medium (YPDA) after cooling the agar to approximately 50°C.

To estimate the antimicrobial activity of basidiomycete isolates against *S. cerevisiae* (Eukaryote), *E. coli* (Gram negative bacteria) and B. subtilis (Gram positive bacteria), a plug of fresh growing mycelium of these fungi was transferred to the centre of 90 mm petri dishes of appropriate medium and incubated for 5-7 days. These plates were then overlaid with 5-10 ml of microbial overlay medium, before secondary incubation. The temperature and incubation period of this further incubation depends on the challenging microorganism. The incubation conditions for *S. cerevisiae* and *B. subtilis* overlays were 48 hrs at 30°C, and for *E. coli* were 24 hours at 37°C. Both fungal growth and the clearing zone diameter were measured. This plate based bioassay was performed in 3 replicates for each tested basidiomycete, each growth medium and each challenging microbe.

References

1- Shiono Y, Akasaka H, Hiramatsu F, Sato K, Murayama T, Ikeda M. Three sesquiterpenoids, fascicularones E, F, and G produced by the fungus Hypholoma fasciculare. Zeitschrift für Naturforschung B. 2005 Aug 1;60(8):880-4.

2- Shiono Y, Matsuzaka R, Wakamatsu H, Muneta K, Murayama T, Ikeda M. Fascicularones A and B from a mycelial culture of Naematoloma fasciculare. Phytochemistry. 2004 Feb 1;65(4):491-6.

3- Shiono Y, Wakamatsu H, Murayama T, Ikeda M. Fascicularones C and D, Tricyclo [5.4. 0.02. 5] undecane Sesquiterpenoids from the Liquid Culture of Naematoloma fasciculare. Zeitschrift für Naturforschung B. 2004 Jan 1;59(1):119-23.

4- Takahashi A, Kusano G, Ohta T, Ohizumi Y, Nozoe S. Fasciculic acids A, B and C as calmodulin antagonists from the mushroom Naematoloma fasciculare. Chemical and Pharmaceutical Bulletin. 1989 Dec 25;37(12):3247-50.

5- Kubo IS, Matsumoto A, Kozuka M, Wood WF. Calmodulin inhibitors from the bitter mushroom Naematoloma fasciculare (Fr.) Karst.(Strophariaceae) and absolute configuration of fasciculols. Chemical and pharmaceutical bulletin. 1985 Sep 25;33(9):3821-5.

6- Shi XW, Li XJ, Gao JM, Zhang XC. Fasciculols H and I, two lanostane derivatives from Chinese mushroom Naematoloma fasciculare. Chemistry & biodiversity. 2011 Oct;8(10):1864-70.

7- Aqueveque P, Becerra J, Palfner G, Silva M, Alarcon J, Anke T, Sterner O. Antimicrobial activity of metabolites from mycelial cultures of chilean basidiomycetes. Journal of the Chilean Chemical Society. 2006 Dec;51(4):1057-60.

8- Ito Y, Kurita H, Yamaguchi T, Sato M, Okuda T. Naematolin, a new biologically active substance produced by Naematoloma fasciculare (Fr.) Karst. Chemical and Pharmaceutical Bulletin. 1967 Dec 25;15(12):2009-10.

9- Backens S, Steffan B, Steglich W, Zechlin L, Anke T. Antibiotika aus Basidiomyceten, XIX Naematolin und Naematolon, zwei Caryophyllan-Derivate aus Kulturen von Hypholoma-Arten (Agaricales). Liebigs Annalen der Chemie. 1984 Jul 12;1984(7):1332-42.

10- Doi K, Shibata T, Nara M, Tsuboyama S, Sakurai T, Tsuboyama K. Structures of naematolin and naematolin B, 1S, 9S-ring-fused caryophyllane sesquiterpenoids. Chemistry Letters. 1986 May 5;15(5):653-6.

11- Doi K, Shibata T, Yokoyama N, Terasawa H, Matsuda O, Kashino S. Structure of naematolin C and naematolin G, novel 4, 8, 11, 11-tetramethyltricyclo [5.4. 0.0 2, 3] undecane sesquiterpenoids. Journal of the Chemical Society, Chemical Communications. 1990(10):725-6.

12- De Bernardi M, Mellerio G, Vidari G, Vita-Finzi P, Fronza G, Kocòr M, St. Pyrek J. Fungal metabolites. IX. Triterpenes from Naematoloma sublateritium. Journal of Natural Products. 1981 May;44(3):351-6.
